# Unravelling the potential of insects for medicinal purposes – A comprehensive review

**DOI:** 10.1016/j.heliyon.2023.e15938

**Published:** 2023-04-29

**Authors:** Shahida Anusha Siddiqui, Chujun Li, Owusu Fordjour Aidoo, Ito Fernando, Moawiya A. Haddad, Jorge A.M. Pereira, Andrey Blinov, Andrey Golik, José S. Câmara

**Affiliations:** a Technical University of Munich Campus Straubing for Biotechnology and Sustainability, Essigberg 3, 94315 Straubing, Germany; b German Institute of Food Technologies (DIL e.V.), Prof.-von-Klitzing Str. 7, 49610 D-Quakenbrück, Germany; c Guangzhou Unique Biotechnology Co., Ltd, 510663, Guangzhou, China; d State Key Laboratory of Biocontrol, School of Life Science, Sun Yat-sen University, Guangzhou, 510006, China; e Department of Biological, Physical and Mathematical Sciences, University of Environment and Sustainable Development, 00233, Somanya, Ghana; f Department of Plant Pest and Diseases, Faculty of Agriculture, Universitas Brawijaya, Malang, 65145, East Java, Indonesia; g Department of Nutrition and Food Processing, Faculty of Agricultural Technology, Al-Balqa Applied University, 19117, Al-Salt, Jordan; h CQM – Centro de Química da Madeira, Universidade da Madeira, Campus da Penteada, 9020-105 Funchal, Portugal; i North Caucasus Federal University, Pushkina Street 1, 355009, Stavropol, Russia; j Departamento de Química, Faculdade de Ciências Exatas e Engenharia, Universidade da Madeira, Campus da Penteada, 9020-105 Funchal, Portugal

**Keywords:** Entomotherapy, Medicine, Diseases, Entomophagy, Consumer acceptance, Mass rearing, Edible insect

## Abstract

Entomotherapy, the use of insects for medicinal purposes, has been practised for centuries in many countries around the world. More than 2100 edible insect species are eaten by humans, but little is known about the possibility of using these insects as a promising alternative to traditional pharmaceuticals for treating diseases. This review offers a fundamental understanding of the therapeutic applications of insects and how they might be used in medicine. In this review, 235 insect species from 15 orders are reported to be used as medicine. Hymenoptera contains the largest medicinal insect species, followed by Coleoptera, Orthoptera, Lepidoptera, and Blattodea. Scientists have examined and validated the potential uses of insects along with their products and by-products in treating various diseases, and records show that they are primarily used to treat digestive and skin disorders. Insects are known to be rich sources of bioactive compounds, explaining their therapeutic features such as anti-inflammatory, antimicrobial, antiviral, and so on. Challenges associated with the consumption of insects (entomophagy) and their therapeutic uses include regulation barriers and consumer acceptance. Moreover, the overexploitation of medicinal insects in their natural habitat has led to a population crisis, thus necessitating the investigation and development of their mass-rearing procedure. Lastly, this review suggests potential directions for developing insects used in medicine and offers advice for scientists interested in entomotherapy. In future, entomotherapy may become a sustainable and cost-effective solution for treating various ailments and has the potential to revolutionize modern medicine.

## 1 Introduction

Entomotherapy is another name for using insects and insect-derived products for therapeutic purposes [1,2]. Insects and their derived products contain natural compounds with a wide range of biological significance, including antibacterial, antifungal, antiviral, anticancer, antioxidant, anti-inflammatory, and immunomodulatory properties [[3], [4], [5], [6]]. Insects are used as live, cooked, ground, infusions, plasters, salves, ointments, and various other ways [6]. Due to these properties, many communities worldwide have used insects for treating illness. For instance, communities in countries like India, China, and Thailand use insects on the advice of local doctors and elders to treat ailments, such as kidney disease, swelling, intestinal disorders, fortified blood, postpartum hemorrhage, lung diseases like asthma and chronic cough, liver and stomach ailments, toothaches, rheumatism, and other conditions. Moreover, some tribes use bedbugs to treat pain and inflammation in the leg fingers caused by nail insertion or other injuries, while mud from the nest of termites is used to treat inflammation in the body. Several studies have also shown that honey, honeybee larvae and pupae are utilized for various health conditions, including gastrointestinal disorders, gastric problems, mental distress, treatment of external wounds, and maggot therapy [[7], [8], [9], [10]].

Entomotherapy has been practised in many countries around the world. According to Wigglesworth [11], many people in Europe throughout the seventeenth century believed that insects had some therapeutic value. These Europeans used insects to treat many health-related complications, such as epilepsy, earaches, scratches, rheumatism and anaemia [12]. Recent research into the antitumoral potential of the Chartergellus-CP1 peptide found in *Chartergellus communis* wasp venom in two different breast cancer cell lines (HR+ and triple-negative) showed encouraging results by killing just cancer cells while leaving healthy cells alone [13]. Blister beetles were used as an aphrodisiac throughout Europe, but recent advances show that they can also reduce pain from kidney stones, urinary tract infections and burns [14]. According to Verma and Prasad [15], these beetles contain cantharidin, which has a protein blocker that fights infections. These proteins can target only the infected cells, making them ideal for use in the immune system's fight against infections. Despite these therapeutic uses of insects and insect-based products, many studies have mainly focussed on their nutritional properties. In contrast to earlier studies, the information in our review offers a more fundamental understanding of the medicinal applications of insects and how they might be used in contemporary medicine. A thorough review of the literature is given, and the history, effects, and opportunities associated with the use of various insect species worldwide are discussed, focusing on papers highlighting the identification of insects to the lowest taxonomic rank possible and their publications in peer-reviewed journals.

In this review, we also discussed many insect species used for medicinal purposes, at which stages these species are utilized, and the impact of these insects on human health. We examine if entomotherapy is met with the same opposition as entomophagy. The earlier ideas of gathering insects, the requirement for industrial manufacturing to create significant amounts of insect-based medication, and what insect mass production would entail are discussed. Our review suggests potential directions for developing insects used in medicine and offers advice for scientists interested in entomotherapy.

## 2 History and evolution of the use of insects for medicinal purposes

Insects in medicine have a long history of application in many societies worldwide by different tribes. Medicinal uses of insects, such as silkworms (*Bombyx mori* L.), date back to at least 3000 years in China. At the same time, honeybees (*Apis mellifera* L.) were first recorded during the Xizho Dynasty (about 1100–771 B.C.). Tao Hongjing's “Mingyi Bielu” (Southern and Northern Dynasties, 420–589 A.D.) expanded “Shennong Bencaojing” to include information on nine additional species of medicinal insects. In his book “Compendium of Materia Medica” (1587), Li Shizhen listed seventy-three different insects used for medical purposes. As a result, 105 bug species were included in the supplementary volume to the “Compendium of Materia Medica” by Zhao Xuemin (Qing Dynasty, 1616–1911 A.D.). According to Robert James, who quoted the Dioscorides, “grasshoppers in a suffumigation relieve under a dysury (difficult micturition), especially as is incident to the female sex”. When insects are bruised and mixed with sugar, they are used to treat ulcers and also serve as dewormers [16]. In some parts of the world, earwigs were used to treat convulsion by first drying, powdering and mixing it with the urine of hare to treat ear complications [16]. Research has shown that the Maya employed maggots for therapeutic purposes 1000 years ago [17]. The lac bug (*Kerria lacca* Kerr.) has been used as medicines since the 3rd century [18].

In some parts of Brazil, ants mixed with sugar and added to coffee or juice was useful in treating diseases associated with vision [19]. The therapeutic uses of insects have been evolving since ancient times [20]. For instance, silkworm pupae were only used for one purpose, that was as feed for livestock [21]. However, they have been recently used in modern medicine [22]. Recent advances in entomotherapy include maggot therapy, which involves the selective removal of necrotic tissue from soft tissue wounds with the insertion of life, disinfected blowfly larvae [23]. There have been recent advancements in the use of bees in apitherapy. Melittin, a key peptide found in bee venom, has shown promise in treating inflammation associated with rheumatoid arthritis and multiple sclerosis. Melittin blocks the expression of genes for inflammation, thereby reducing pain. Apitherapy has also provided more insight into its application to treat diseases, like Parkinson's disease by analysing the effects of propolis and royal jelly on the disease [24].

At present, there are about 1000 insects that have been documented to have medicinal properties in different countries worldwide, and includes Africa, India, Japan, Korea, South America, Spain, Tibet and Turkey [25,26]. However, out of the 1000 insects in medicine, about half of them from 70 genera, 63 families and 14 orders have been reported from China. In the Tibetan region of China, eleven insects, including flies, ants, butterflies, cicadas, and four kinds of beetles, such as diving beetles and blister beetles, were identified as insects with medicinal properties [27]. Apart from China, at least 50 different human diseases and conditions had been linked to the use of 50 different insect species from 28 families and 11 orders, have been recorded from India [28].

**Fig. 1:**
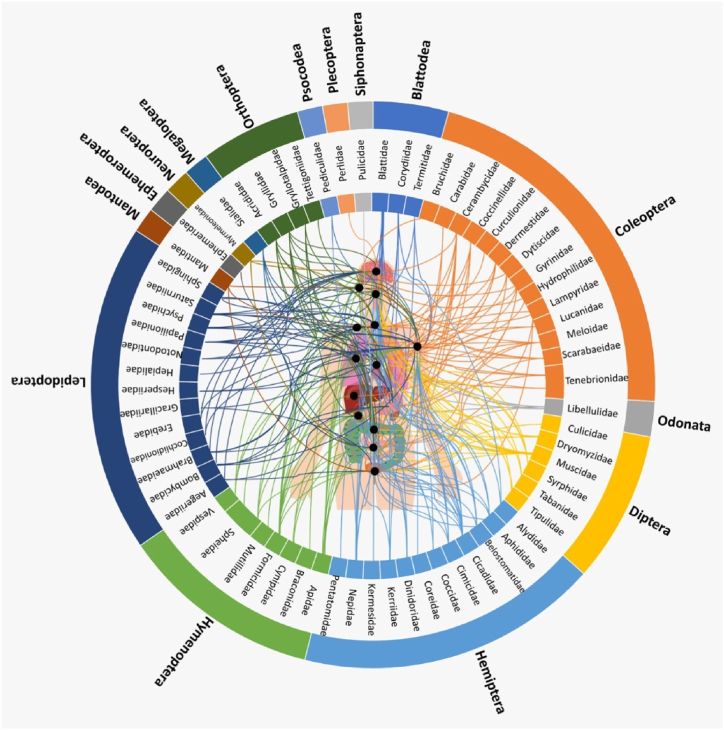
The use of various types of insects for medicinal purposes.

**Fig. 2:**
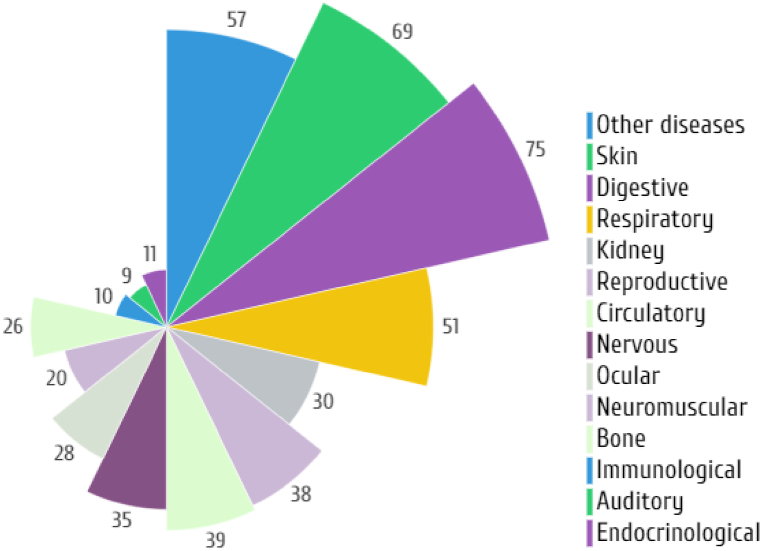
Number of insect species to alleviate diseases.

A large number of insect species belonging to different orders, such as Blattodea, Coleoptera, Diptera, Odonata, Hemiptera, Hymenoptera, Lepidoptera, Mantodea, Orthoptera, that have been useful in the treatment of various diseases are presented in Table 1 and illustrated in Fig. 1. It is worth mentioning that most of the identified medicinal insect were used to treat digestive and skin diseases. However, the records of application methods (e.g., oral, external applied) are limited, therefore, more detail in application description is needed in future studies on medicinal insect used by indigenous tribes across the globe. In more detail, the number of insect species used in the treatment of various diseases is shown in Fig. 2.

**Table 1:** Various insect species and their records of medicinal uses.

Order	Family	Scientific name	Therapeutic benefits	Ways of utilization	References
**Blatodea**	**Blattidae**	*Periplaneta americana* (Linnaeus)	Burning, gastroenteritis, earache, rectal prolapse, shingles, skin, stomach disorders, consitipation, heartburn, colic; whooping cough, boils, dropsy, wart, Bright's disease, ulcers, stimulate lactation, anti-tumor, whooping cough, difficulty urinating, renal colic, and asthma	Oral	[1,12,16,19,[29], [30], [31]]
		*Periplaneta fuliginosa* (Serville)	Skin and stomach disorders	Non-specified	[12,16,19,26,31,32]
		*Blattella germanica* (Linnaeus)	Skin and stomach disorders	Non-specified	[26]
		*Blatta orientalis* (Linnaeus)	Skin, stomach disorders, tetanus and ear pain, anti-asthmatic, anti-anaphylactic properties dropsy, pleurisy, and pericarditis	Non-specified	[26]
	**Corydiidae**	*Eupolyphaga sinensis* (Walker)	Ischemic heart disease, cardiac function; hepatic diseases, gynecopathy, and atherosclerosis and epilepsy		[12,16,19,31]
		*Polyphaga plancyi* (Bolívar)	Menstrual problems, fracture, amenorrhea	Non-specified	[19]
	**Termitidae (termites)**	*Microcerotermes exiguus* (Hagen)	Asthma, bronchitis, influenza, whooping cough, and flu	Non-specified	[[33], [34], [35]]
		*Nasutitermes corniger* (Motschulsky)	Asthma, cough, flu, and sore throat	Non-specified	[34]
		*Nasutitermes macrocephalus* (Silvestri)	Asthma, Leakage, Bronchitis, ‘catarrh in the chest’ coughs, influenza, sore throat, sinusitis, tonsillitis, and hoarseness	Non-specified	[34]
		*Odontotermes feae* (Wasmann)	Asthma	Oral	[36]
		*Macrotermes bellicosus* (Smeathman)	Suture wounds	Non-specified	[37]
		*Odontotermes formosanus* (Shiraki)	Ulcer, Better health, Body pain, Rheumatics, Anemia and Enhancement of lactation	Non-specified	[33]
		*Macrotermes* sp*.*	Sexual impotence, inflammation, dislocation, congenita malformation, headache, vomiting, diarrhea, articular pain, bone pain, sprain, general fatigue, fracture, gonorrhea, and child malnutrition	Topical and Oral	[38,39]
		*Pseudacanthotermes spiniger* (Sjoestedt)	Fungus and bacterial infection	Non-specified	[39]
		*Nasutitermes* sp.	Inflammation	Topical	[39]
		*Microtermes obesi* (Holmgren)	Liver disorder	Oral	[25]
		*Trinervitermes* sp.	Mumps, burn, fracture, iron deficiency, dropsy, inflammation, edemas, wound and vomiting	Topical	[39]
**Coleoptera**	**Bruchidae**	*Pachymerus nucleorum* (Fabr.)	Earache	Non-specified	[40]
	**Cicindelidae**	*Cicindela chinensis* (DeGeer)	Skin, tumours and gynaecological problems	Non-specified	[26]
		*Pheropsophus* spp.	Alcoholism	Oral	[29]
	**Cerambycidae**	*Apriona rugicollis* (Chevrolat)	Lung problems, cramps, and palsy	Non-specified	[26]
		*Batocera rubus* (Linnaeus)	Analgesic and gastro-intestinal problems, treating malaria, typhoid and aphrodisiac	Oral	[28]
		*Batocera parryi* (Hope)	Analgesic and gastro-intestinal problems, treating malaria, typhoid, and aphrodisiac	Oral	[28]
		*Batocera rufomaculata* (De Geer)	Analgesic and gastro-intestinal problems, treating malaria, typhoid, and aphrodisiac	Oral	[28]
		*Chloridolum thaliodes* (Bates)	Treating smallpox	Non-specified	[26]
		*Batocera lineolata* (Chevrolat)	Mitigate cramps, cancer therapy and diphtheria, smallpox	Non-specified	[26]
		*Orthosoma brunneum* (Forster)	Analgesic and gastro-intestinal problems, treating malaria, typhoid, and aphrodisiac	Oral	[28]
		*Aromia moschata* (Linnaeus)	Vesicatory and acted like cantharides	Non-specified	[16]
	**Coccinellidae**	*Coccinella septempunctata* (Linnaeus)	Wound	Non-specified	[39]
	**Curculionidae**	*Larinus maculatus* (Gyllenhal)	Respiratory organs	Non-specified	[16]
		*Brachycerus ornatus* (Drury)	Stomach pains	Non-specified	[8,41]
	**Dermestidae**	*Ips typographus* (Linnaeus)	Vesicators and opening abscesses	Non-specified	[16]
	**Dytiscidae**	*Cybister brevis* (Aubé)	Asthma, respiratory and stomach problems	Non-specified	[26]
		*Cybister chinensis* (Motschulsky)	Asthma, respiratory and stomach problems	Non-specified	[26]
		*Cybister tripunctatus* (Olivier)	Asthma, respiratory and stomach problems	Non-specified	[26]
		*Rhantus pulverosus* (Stephens)	Skin disorders	Non-specified	[26]
	**Gyrinidae**	*Gyrinus curtus* (Motschulsky)	Lung and stomach problems, fever, and cramps	Non-specified	[26]
		*Gyrinus japonicus* (Sharp)	Lung and stomach problems, fever, and cramps	Non-specified	[26]
		*Dineutus marginatus (*Sharp)	Lung and stomach problems, fever, and cramps	Non-specified	[26]
	**Hydrophilidae**	*Sternolophus rufipes* (Fabricius)	Skin disorders, cramps, and whooping cough	Non-specified	[26]
		*Hydrophilus affinis* (Thunberg)	Skin disorders, cramps, and whooping cough	Non-specified	[26]
		*Hydrophilus acuminatus* (Motschulsky)	Skin disorders, cramps, and whooping cough	Non-specified	[26]
	**Lampyridae**	Lampyridae spp.	Cancer	Oral	[29]
		*Aquatica lateralis* (Motschulsky)	Bleedings, tumours, whooping cough, haemorrhoids, and as hair tonic	Non-specified	[26]
	**Lucanidae**	*Lucanus macrifemoratus* (Motschulsky)	Treatments of gynaecological problems	Non-specified	[26]
		*Prosopocoilus inclinatus* (Motschulsky)	Treatments of gynaecological problems	Non-specified	[26]
	**Meloidae**	*Epicauta gorhami* (Marseul)	Treatments of hair, skin excretory (kidney) system, rabies and warts	Non-specified	[25]
		*Mylabris pustulata* (Latreille)	Dog bite and Hydrophobia	Non-specified	[26]
		*Mylabris* sp.	Blisters and warts	Topical	[25,28]
		*Lytta vesicatoria* (Linnaeus)	Urinary disorders and aphrodisiac	Non-specified	[8,42]
		*Berberomeloe majalis* (Linnaeus)	Warts	Non-specified	[16]
		*Pseudomeloe andensis* Guérin-Méneville	Warts	Non-specified	[16]
		*Palembus dermestoides* (Farmaire)	Sexual impotence, ophthalmological problems, rheumatism, and weakness	Non-specified	[8,43]
		*Lytta* sp.	Sickle cell anemia	Oral	[39]
	**Scarabaeidae**	*Melolontha vulgaris* (Fabricius)	Scratches, anemia, and rheumatism	Non-specified	[8,12]
		*Scarabaeus laticollis* (Linnaeus)	Painful urination	Non-specified	[39]
		*Propomacrus* sp.	Cough	Non-specified	[1]
		*Strategus aloeus* (Linnaeus)	Aphrodisiac	Non-specified	[8]
		*Megasoma actaeon* (Linnaeus)	Aphrodisiac	Non-specified	[8]
	**Tenebrionidae**	*Alphitobius diaperinus* (Panzer)	Diabetes and obesity	Non-specified	[44,45]
		*Palembus dermestoides* (Farmaire)	Asthma, arthritis, tuberculosis and sexual impotence	Non-specified	[46]
		*Blaps sulcata* (Laporte de Castelnau)	Scorpion bites	Non-specified	[16]
	Tenebrionidae	*Tenebrio molitor* (Linnaeus)	Anti-inflammatory (stroke)	Non-specified	[47]
**Odonata**	**Libellulidae**	*Sympetrum darwinianum* (Selys)	Throat aches, asthma, tumours, fever, and whooping cough	Non-specified	[26]
		*Sympetrum pedemontanum* (Müller in Allioni)	Asthma	Non-specified	[26]
		*Sympetrum croceolum* (Selys)	Asthma	Non-specified	[26]
		*Sympetrum frequens* (Sélys)	Asthma	Non-specified	[26]
		*Orthetrum albistylum* (Selys)	Asthma	Non-specified	[26]
		*Crocothemis servilia* (Drury)	Ear, eye, throat and gut problems, fever, diphtheria, and cough	Non-specified	[26]
**Diptera**	**Culicidae**	*Culex pipiens* (Linnaeus)	Venereal diseases	Non-specified	[26]
		*Aedes japonicus* (Theobald)	Venereal diseases	Non-specified	[26]
		*Aedes albopictus* (Skuse)	Venereal diseases	Non-specified	[26]
		*Anopheles japonicus* Coluzzi	Venereal diseases	Non-specified	[26]
	**Dryomyzidae**	*Dryomyza formosa* Wiedemann	Fever, snake bite, gut and stomach problems and vision	Non-specified	[26]
	**Muscidae**	*Fannia canicularis* (Linnaeus)	Snake bites, fever, gut and stomach problems, vision, tooth ache, skin disorders and haemorrhoids	Non-specified	[26]
		*Musca domestica* (Linnaeus)	Sickle cell anaemia, male infertility, eye cysts, baldness, scorpion and snake bites, fever, gut and stomach problems, vision Fever, tooth ache, skin disorders and haemorrhoids	Oral	[26,39]
		*Muscina stabulans* (Fallen)	Snake bites, fever, gut and stomach problems and vision tooth ache, skin disorders and haemorrhoids	Non-specified	[26]
		*Fannia canicularis* (Linnaeus)	Snake bites, fever, gut and stomach problems and vision tooth ache, skin disorders and haemorrhoids	Non-specified	[16,26,39]
		*Calliphora lata* (Coquillett)	Snake bites, fever, gut and stomach problems, vision, tooth ache and skin disorders and venereal diseases	Non-specified	[26]
	**Syrphidae**	*Eristalis tenax* (Linnaeus)	Vision, tooth ache, fever, and cramps	Non-specified	[26]
	**Tabanidae**	*Tabanus trigonus* Coquillett	Vision and tumours	Non-specified	[26]
		*Tabanus rufidens* (Bigot)	Vision and tumours	Non-specified	[26]
		*Tabanus chrysurus* (Loew)	Vision and tumours	Non-specified	[26]
		*Tabanus mandarinus* (Schiner)	Vision and tumours	Non-specified	[26]
	**Tipulidae**	*Tipula oleracea* (Linnaeus)	Analgesic and measles in children	Oral	[28]
**Hemiptera**	**Alydidae**	*Leptocorisa varicornis* (Fabricius)	Fever	Oral	[29]
	**Aphididae**	*Schlechtendalia chinensis* (Bell)	Eggs used in connection with bleedings, intestinal and uterine problems; adults in connection with cough, dysentery, and haemorrhoids	Non-specified	[26]
	**Belostomatidae**	*Lethocerus deyrollei* (Vuillefroy)	Eggs used in connection with bleedings, intestinal and uterine problems; adults in connection with cough, dysentery, and haemorrhoids	Non-specified	[26]
		*Lethocerus indicus* (Lepeletier and Serville)	Nocturnal emission, gastro-intestinal problems, rheumatoid arthritis, and wound healing	Oral	[1,28]
	**Cicadidae**	*Terpnosia vacua* (Kato)	Anaemia; ear problems, tooth ache, fever as well as kidney problems, tumours, smallpox, coughs, and haemorrhoids; Migraine headache and ear infection	Non-specified	[26]
		*Platypleura kaempferi* (Fabricius)	Anaemia; ear problems, tooth ache, fever as well as kidney problems, tumours, smallpox, coughs, and haemorrhoids	Non-specified	[26]
		*Graptopsaltria nigrofuscata* (Motschulsky)	Anaemia; ear problems, tooth ache, fever as well as kidney problems, tumours, smallpox, coughs, and haemorrhoids	Non-specified	[26]
		*Cryptotympana japonensis* (Kato)	Anaemia; ear problems, tooth ache, fever as well as kidney problems, tumours, smallpox, coughs, and haemorrhoids	Non-specified	[26]
		*Huechys sanguinea* (DeGeer)	Migraine headaches and ear infections	Non-specified	[8]
		*Tanna japonensis* (Distant)	Anaemia; ear problems, tooth ache, fever as well as kidney problems, tumours, smallpox, coughs, and haemorrhoids	Non-specified	[26]
		*Hyalessa maculaticollis* (Motschulsky)	Anaemia; ear problems, tooth ache, fever as well as kidney problems, tumours, smallpox, coughs, and haemorrhoids	Non-specified	[26]
		*Meimuna opalifera* (Walker)	Anaemia; ear problems, tooth ache, fever as well as kidney problems, tumours, smallpox, coughs, and haemorrhoids	Non-specified	[26]
	**Cimicidae**	*Cimex lectularius* Linnaeus	Venom of snakes, lethargy, urinary problems, eyes, ears, hysterical suffocation, worms, and epileptic attacks	Non-specified	[16]
	**Coccidae**	*Ericerus pela* (Chavannes)	Bleedings, lung and stomach problems and warts	Non-specified	[26]
	**Coreidae**	*Thasus gigas* (Klug)	Diabetes	Oral	[28,48]
	**Dinidoridae**	*Coridius singhalanus* (Distant)	Fever, treating jaundice, malaria and to increase milk production.	Non-specified	[45]
	**Kerriidae**	*Kerria lacca* (Kerr)	Diarrhea, indigestion, measles, macula, and scabies	Non-specified	[6,49]
	**Kermesidae**	*Kermes ilicis* (Linnaeus)	To prevent abortion from strain and injury, and menstrual problems	Non-specified	[16]
	**Nepidae**	*Laccotrephes japonensis* (Scott)	Eggs used in connection with bleedings, intestinal and uterine problems; adults in connection with cough, dysentery and haemorrhoids	Non-specified	[26,50]
		*Ranatra chinensis* (Mayr)	Eggs used in connection with bleedings, intestinal and uterine problems; adults in connection with cough, dysentery, and haemorrhoids	Non-specified	[26]
		*Ranatra unicolor* (Scott)	Eggs used in connection with bleedings, intestinal and uterine problems; adults in connection with cough, dysentery, and haemorrhoids	Non-specified	[26]
		*Laccotrephes ruber* (Linnaeus)	Cardiovascular (blood purification)	Oral	[28]
	**Pentatomidae**	*Udonga montana* (Distant)	Pain	Oral	[28]
**Hymenoptera**	**Apidae**	*Apis cerana indica* (Fabricius)	Cough, fever, cancer, cracks, diabetes and scars, cold, sore throat, burns, tongue ulcer, gastritis, and wart	Oral	[1,6,25,29,51,52]
		*Apis cerana japonica* (Radoszkowski)	Skin, respiratory, urinary, and intestinal disorders, snake bite and rabies; skin and digestive problems and snake bite: Larvae and adults in connection with rheumatism, influenza, the common cold and whooping cough; wax for freckles and constipation	Oral	[26]
		*Apis dorsata* Fabricius	Cracks and scars, skin, respiratory, urinary, and intestinal disorders, snake bite and rabies, skin and digestive problems, rheumatism, influenza, common cold, whooping cough; wax for freckles and constipation, cold, cough and sore throat, burns, tongue ulcer, gastritis, anti-inflammatory, anti-nociceptive, and anti-arthritic properties.	Oral	[6,25,26,51]
		*Apis florea* (Fabricius)	Respiratory problems (coughs), cold, sore throat, burns, tongue ulcer, gastritis, and wart	on-specified	[28,51]
		*Apis laboriosa* (Smith)	Respiratory problems (coughs)	on-specified	[28]
		*Apis mellifera* (Linnaeus)	Throat pain, irregular menstruation, cough, cold, general fatigue, sickle cell anemia, and burns and cuts, menopausal problems, Intestinal, helminthiasis, strangulated hernia, sexual impotence, insomnia, memory losss, heart diseases, difficulty breathing, voice extinction, pneumonia, bladder lithiasis, diabetes, constipation, hemorrhage in women, nausea, burns, pyrosis, toxin, stomach aches, foot pain, gonorrhea, ulcer, itching, anal bleeding, amenorrhea and infertility	on-specified	[8,25,53,54]
		*Lepidotrigona arcifera* (Cockerell)	Gynaecological/andrological problems, and venomous animal bites	on-specified	[28,51]
		*Lophotrigona canifrons* (Smith)	Gynaecological/andrological problems, and venomous animal bites	on-specified	[28,51]
		*Melipona indecisa* (Cockerell)	Sour throat	on-specified	[55]
		*Melipona mimetica* (Cockerell)	Balm, blood kidney, eyes, inflammation and sour	on-specified	[55]
		*Melipona scutellaris* (Latreille)	Cough	on-specified	[8]
		*Nannotrigona perilampoides* (Cresson)	Eye	on-specified	[55]
		*Paratrigona eutaeniata* Camargo et Moure	Eyes	on-specified	[55]
		*Scaptotrigona ederi* Engel	Balm, kidney, eyes, inflammation sour throat, tumor, wound healing	on-specified	[55]
		*Trigona spinipes* (Fabricius)	Cough	on-specified	[8]
		*Xylocopa appendiculata* (Smith)	Fever, respiratory/lung ailments, and haemorrhoids	on-specified	[26]
	**Braconidae**	*Euurobracon penetrator* (Smith)	Cases of cramp	on-specified	[26]
	**Cynipidae**	*Diplolepis rosae* (Linnaeus)	Diarrhea and dysentery, and for scurvy, stone and worms	on-specified	[16]
	**Formicidae**	*Pogonomyrmex californicus* (Buckley)	Panacea	Oral	[56]
		*Tetraponera rufonigera* (Jordan)	Body pain	Oral	[29]
		*Oecophylla smaragdina* (Fabricius)	Coughs, fever, gastritis, malaria, typhoid, edema, sinus infections, analgesic, common cold, Jaundice, enteric problems, whooping hungriness, cancer and nose bleeding, malaria, throat pain, breathing problem, asthma, boils/pox, measles, for the treatment of detoxification blood, arresting hemorrhage during miscarriages, restoration of uterus, removal of any aftermath from the uterine canal after childbirth, stimulating pulse and heartbeat, and dizziness	Oral	[6,25,28,29,51,[57], [58], [59], [60]]
		*Myrmicaria brunnea* (Saunders)	Body ache	Oral	[29]
		*Pseudoneoponera rufipes* (Jerdon)	Scabies, toothache, wounds, high blood pressure and malaria	Non-specified	[25]
		*Polyrhachis dives* Smith	Rheumatoid, osteoarthritis, inflammatory diseases, and central nervous system	Oral	[61]
		*Camponotus maculatus* (Fabricius)	Azoospermia	Oral	[39]
		*Pseudoneoponera rufipes* (Jerdon)	Toothaches and blood pressure	Non-specified	[6]
		*Tetramorium* sp	Anti-bacterial properties, sprain, Inflammation, cyst, hip pain, headache, neurological problems, retention of acute urinary, gynecological problems, and chronic cough	Topical and Oral	[39]
		*Camponotus* sp.	Foot pain and retention of acute urinary	Topical and Oral	[39]
		*Pachycondyla* sp.	Knee pain, headache, stomach aches, neurological problems, retention of acute urinary and toxin	Topical and Oral	[39]
	**Mutillidae**	*Dasymutilla ocidentalis* (Linnaeus)	Chickenpox	Non-specified	[62]
	**Sphecidae**	*Sceliphron* sp.	Inflammation, vomiting, allergy due to stings, sprain, hiccups, female infertility, lipoma, soa throat, hip pain, foot pain, mumps, cough, fontanel problem, vomiting and migraine	Non-specified	[25]
	**Vespidae**	*Vespula vulgaris* (Linnaeus)	Lipoma, heart diseases and whitlow	Topical and Oral	[39]
		*Polistes carolina* (Linnaeus)	Piles and general wound	Non-specified	[25]
		*Vespa affinis* (Linnaeus)	Cancer	Oral	[29]
		*Vespa mandarinia* (Smith)	Skin diseases, fever respiratory problems, whooping cough, ear, eye and dental problems, skin disorders and cramps	Non-specified	[26]
		*Vespa auraria* (Smith)	Skin diseases, fever, respiratory problems, whooping cough, ear, eye and dental problems, cramps, and haemorrhoids	Non-specified	[26]
**Lepidoptera**	**Aegeriidae**	*Paranthrene regalis* (Butler)	Stomach upsets, cramps, gynaecological issues, and diphtheria	Non-specified	[26]
	**Bombycidae**	*Bombyx mori* (Linnaeus)	Pneumonia, stopping bleedings, throat troubles, fever, and snake bite Pupae used in connection with throat problems, tuberculosis, kidney problems, bleedings, counter snake bite, vertigo and convulsions and fever	Non-specified	[16,25,26]
	**Brahmaeidae**	*Brahmaea japonica* (Butler)	Cramps, respiratory, anemia and stomach troubles	Non-specified	[26]
	**Cochlidionidae**	*Cnidocampa flavescens* (Walker)	Cramps, vision	Non-specified	[26]
	**Erebidae**	*Euproctis chrysorrhoea* (Linnaeus)	homeopathic tinctures	Non-specified	[16]
		*Spilosoma obliqua* (Walker)	Dog bites	Non-specified	[6]
	**Gracillariidae**	*Stomphastis thraustica* (Meyrick)	Fever and to increase milk flow in lactating women	Non-specified	[6,63]
	**Hesperiidae**	*Erionota torus* (Evans)	Sexual weakness and venomous animal bites	Oral	[28]
	**Hepialidae**	*Endoclita excrescens* (Butler)	Lung and stomach troubles and snake bite	Non-specified	[26]
	**Notodontidae**	*Bombyx processionea* (Linnaeus)	Homeopathic tinctures	Non-specified	[16]
	**Papilionidae**	*Pachliopta aristolochiae* (Fabricius)	Snake bite	Non-specified	[25]
		*Holocerus vicarious* Karsch	Fever and cramps	Non-specified	[26]
		*Papilio xuthus* (Linnaeus)	Fever and cramps, skin disorders, lumps, and tumours	Non-specified	[26]
		*Papilio machaon* (Linnaeus)	Fever and cramps, skin disorders, lumps, and tumours	Non-specified	[26]
		*Papilio protenor* (Cramer)	Fever and cramps, skin disorders, lumps, and tumours	Non-specified	[26]
		*Papilio macilentus* (Janson)	Fever and cramps, skin disorders, lumps, and tumours	Non-specified	[26]
		*Byasa alcinous* (Klug)	Fever and cramps, skin disorders, lumps, and tumours	Non-specified	[26]
		*Graphium sarpedon nipponus* (Fruhstorfer)	Fever and cramps, skin disorders, lumps, and tumours	Non-specified	[26]
	**Psychidae**	*Cryptothelea minuscula* (Butler)	Toothache and respiratory problems	Non-specified	[26]
		*Oiketicus kirbyi* (Guilding)	Asthma, earache, and hemorrhage	Non-specified	[6]
	**Saturniidae**	*Antheraea yamamai* (Guérin-Méneville)	Asthma, cramps, throat and skin troubles, lumps, and cramps	Non-specified	[26]
		*Antheraea pernyi* (Guérin-Méneville)	Tumor growths and lumps	Non-specified	[26]
		*Samia cynthia* (Drury)	Analgesic, blood pressure and diabetes	Non-specified	[25]
		*Caligula japonica* (Moore)	Skin problems	Non-specified	[26]
		*Cirina butyrospermi* (Vuillet)	Asthma, arteria, hypertension, avitaminosis, abdominal bloating, diabetes, and tetanus	Oral	[39]
		*Rhodinia fugax* (Butler)	Whooping cough	Non-specified	[26]
	**Sphingidae**	*Deilephila elpenor* (Linnaeus)	Tuberculosis, stomach upsets, lumps, tumours and fever	Non-specified	[26]
		*Agrius convolvuli* (Linnaeus)	Tuberculosis, stomach upsets, lumps, tumours and fever	Non-specified	[26]
		*Psilogramma increta* (Walker)	Tuberculosis, stomach upsets, lumps, tumours and fever	Non-specified	[26]
		*Theretra nessus* (Drury)	Tuberculosis, stomach upsets, lumps, tumours and fever	Non-specified	[26]
		*Theretra oldenlandiae* (Fabricius)	Tuberculosis, stomach upsets, umps, tumours and fever	Non-specified	[26]
		*Macroglossum stellatarum* (Linnaeus)	Tuberculosis, stomach upsets, umps, tumours and fever	Non-specified	[26]
**Mantodea**	**Mantidae**	*Hierodula coarctata* (Saussure)	Urological problems (enuresis)	Oral	[28]
		*Mantis religiosa* (Linnaeus)	Otorrhoea, fever, beriberi, tooth ache, fever, hair, and respiratory problems	Topical	[26,29]
		*Tenodera sinensis* (Saussure)	Otorrhoea, fever, beriberi, tooth ache, warts, fever, hair and respiratory problems	Masticate on warts	[26,28]
		*Tenodera angustipennis* (Saussure)	Otorrhoea, fever, beriberi, tooth ache, fever, hair, and respiratory problems	Non-specified	[26]
		*Statilia maculata* (Thunberg)	Otorrhoea, fever, beriberi, tooth ache, fever, hair, and respiratory problems	Non-specified	[26]
		*Hierodula patellifera* (Serville)	Otorrhoea, fever, beriberi, tooth ache, fever, hair, and respiratory problems	Non-specified	[26]
**Ephemeroptera**	**Ephemeridae**	*Ephemera danica* Müller	Stomach disturbance	Non-specified	[26]
**Neuroptera**	**Myrmeleonidae**	*Hagenomyia micans* (McLachlan)	Fever, migraine/headaches, beriberi, gonorrhea, and whooping cough	Non-specified	[26]
**Megaloptera**	**Sialidae**	*Protohermes grandis* (Thunberg)	Lung, stomach, and gut problems	Non-specified	[26]
**Orthoptera**	**Acrididae**	*Oxya* sp.	Nocturnal emission	Oral	[1]
		*Oxya velox* (Fabricius)	Adults used in cases of fever, respiratory, skin, and gynaecological problems, effective in treating cancer, haemorrhoids and anaemia	Non-specified	[26]
		*Oxya vicina* Wattenwyl.	Fever, respiratory, skin, gynaecological problems, cancer, haemorrhoids and anaemia	Non-specified	[26]
		*Acrida bicolor* (Thunber)	Hypertention	Non-specified	[8,38]
		*Hieroglyphus banian* (Fabricius)	Dog bite	Non-specified	[25]
		*Locusta migratoria* (Linnaeus)	Effective antidote to scorpion bites, piles, and thirst	Non-specified	[16]
		*Schistocerca gregaria* (Forsskål)	Wound	Topical	[39]
		*Melanoplus* sp.	Gastrointestinal problems	Oral	[28]
	**Gryllidae**	*Tarbinskiellus portentosus* (Lichtenstein)	Malaria, headaches, and gastro-intestinal problems	Oral	[28]
		*Gryllus assimilis* (Fabricius)	Urine retention	Non-specified	[8,62]
		*Acheta domesticus* (Linnaeus)	Pain, deafness, eyesight, and pancreas health	Oral	[29,39]
	**Gryllotalpidae**	*Scapteriscus borellii* (Giglio-Tos)	Intestinal worms	Oral	[29]
		*Gryllotalpa africana* (Palisot de Beauvois)	Fever, mitigate skin and kidney troubles, fight tumor growths and venereal disease	Non-specified	[26]
	**Tettigoniidae**	*Tettigonia verrucivora* (Kirby)	Warts	Non-specified	[16]
**Psocodea**	**Pediculidae**	*Pediculus humanus* (Linnaeus)	Jaundice, venereal diseases	Non-specified	[26,64]
**Plecoptera**	**Perlidae**	*Perla tinctipennis* (McLachlan)	Cramps	Non-specified	[28]
		*Perla tibialis* (Pictet)	Cramps	Non-specified	[28]
**Siphonaptera**	**Pulicidae**	*Pulex irritans* (Linnaeus)	Venereal diseases	Non-specified	[26]
		*Ctenocephalides canis* (Curtis, 1826)	Venereal diseases	Non-specified	[26]
		*Ctenocephalides felis* (Bouché, 1835)	Venereal diseases	Non-specified	[26]

## 3 Effects and consequences of using medicinal insects

### 3.1 Insect species used for medicinal purposes and their associated stage being used

In total, 235 valid species were documented in several literatures that summarized insects used in folk medicine, which include insects from China [65,66], India [28], Africa [39], and Latin America [63]. Table 2 listed all the 235 species from 15 different orders, within which Hymenoptera contains the largest medicinal insect species count (62 species), followed by Coleoptera (47), Orthoptera (28), Lepidoptera (23), and Blattodea (21). The other orders contain much less (e.g., ≤11) species, which sum up to 55. At the family level, Apidae (27) contains the largest medicinal insect species documented, followed by Vespidae (19), Formicidae (15), Gryllidae (11), Cerambycidae (10), Meloidae (9), Termitidae (9), Acrididae (8), Libellulidae (8), Cicadidae (8), and Mantidae (7), which sum up to 50% of the 235 species documented. Some genera contain more than one medicinal insect species. For example, seven species were reported in genus *Melipona*, seven species were reported in genus *Vespa*, and another seven species were reported in genus *Apis*.

**Table 2:** Insect species recorded in folk medicine and the stage and ingredient used.

Order	Family	Species	Stage or ingredient used	Reference
**Blattodea**	**Blaberidae**	Epilampra sp.	Nymph/Adults	[28]
		Opisthoplatia orientalis (Burmeister)	Nymph/Adults	[66]
		Rhyparobia maderae (Fabricius)	Non-specified	[63]
	**Blattidae**	*Blatta orientalis* L.	Non-specified	[66]
		*Blattella germanica* L.	Nymphs/Adults	[66]
		Eurycotis manni Rehn	Nymph	[63]
		*Periplaneta americana* L.	Adults	[39,63,66]
		Periplaneta australasiae (Fabricius)	Non-specified	[66]
	**Corydiidae**	Eupolyphaga sinensis (Walker)	Nymph/Adults	[66]
		Eupolyphaga yunnanesis (Chopard)	Nymph/Adults	[66]
	**Rhinotermitidae**	Coptotermes formosanus Shiraki	Adults	[66]
		Reticulitermes flaviceps Oshima	Adults	[66]
	**Termitidae**	Macrotermes annandalei (Silvestri)	Larvae/Adults	[66]
		Macrotermes barneyi Light	Non-specified	[65,66]
		*Macrotermes* sp.	Adults/Nest	[28,39]
		Microcerotermes exiguus (Hagen)	Non-specified	[63]
		Nasutitermes corniger (Motschulsky)	Non-specified	[63]
		Nasutitermes macrocephalus (Silvestri)	Non-specified	[63]
		*Nasutitermes* sp.	Nest	[39]
		Odontotermes formosanus (Shiraki)	Non-specified	[66]
		*Trinervitermes* sp.	Adults, Nest	[39]
**Coleoptera**	**Buprestidae**	Chalcophora japonica Gory	Adults	[65]
	**Carabidae**	Pheropsophus jessoensis (A.Morawitz)	Non-specified	[65,66]
	**Cerambycidae**	Anoplophora chinensis (Forster)	Larvae/Adults	[66]
		Anoplophora glabripennis (Motschulsky)	Larvae	[65]
		Apriona germari Hope	Larvae/Adults	[65,66]
		Aromia bungii (Faldermann)	Larvae	[65]
		Batocera horsfieldi (Hope)	Adults	[66]
		Batocera parryi Hope	Larvae	[28]
		Batocera rubus L.	Larvae	[28]
		Batocera rufomaculata (De Geer)	Larvae	[28]
		Macrodontia cervicornis (L.)	Non-specified	[63]
		Orthosoma brunneum (Forster)	Larvae	[63]
	**Chrysomelidae**	Coraliomela brunnea (Thunberg)	Non-specified	[63]
		Pachymerus nucleorum (Fabricius)	Non-specified	[63]
	**Coccinellidae**	*Coccinella septempunctata* L.	Adults	[39]
	**Curculionidae**	Rhina barbirostris Champion	Non-specified	[63]
		Rhinostomus barbirostris (Fabricius)	Non-specified	[63]
		Rhynchophorus palmarum L.	Non-specified	[63]
	**Dytiscidae**	Cybister japonicus Sharp	Adults	[65]
		Cybister limbatus (Fabricius)	Adults	[28]
		Cybister tripunctatus lateralis (Fabricius)	Adults	[28]
	**Elateridae**	Pleonomus canaliculatus (Faldermann)	Non-specified	[66]
	**Gyrinidae**	Gyrinus curtus Motschulsky	Non-specified	[66]
	**Hydrophilidae**	Hydrophilus caschmirensis Redtenbacher	Adults	[28]
	**Lampyridae**	Luciola ficta Olivier	Larvae/Adults	[66]
	**Meloidae**	Epicauta hirticornis Haag-Rutenberg	Non-specified	[66]
		Lytta caraganae (Pallas)	Non-specified	[66]
		Lytta sp.	Adults	[39]
		Meloe coarctatus Motschulsky	Non-specified	[66]
		Mylabris cichorii L.	Adults	[66]
		Mylabris phalerata (Pallas)	Non-specified	[66]
		Mylabris sp.	Adults	[28]
		Palembus dermestoides (Fairmaire)	Non-specified	[63]
		Pseudomeloe andensis (Guérin-Méneville)	Non-specified	[63]
	**Melolonthidae**	Holotrichia diomphalia Bates	Larvae, Adults	[65,66]
		Holotrichia morosa Waterhouse	Larvae/Adults	[66]
		Holotrichia oblita (Faldermann)	Larvae/Adults	[66]
		Polyphylla gracilicornis (Blanchard)	Adults	[65]
	**Rutelidae**	Anomala corpulenta Motschulsky	Larvae/Adults	[66]
	**Scarabaeidae**	Allomyrina dichotoma L.	Larvae	[65,66]
		Potosia (Liocola) brevitarsis (Lewis)	Larvae	[65]
		Potosia (Liocola) brevitarsis (Lewis)	Larvae/Adults	[66]
		*Scarabaeus laticollis* L.	Rolled dung	[39]
		Geotrupes laevistriatus Motschulsky	Adults	[66]
		Geotrupes substriatellus L.	Adults	[66]
	**Tenebrionidae**	Martianus dermestoides (Chevrolat)	Non-specified	[66]
		*Tenebrio molitor* L.	Chitin	[65,66]
**Dermaptera**	**Forficulidae**	Forficula auricularia L.	Non-specified	[63]
**Diptera**	**Calliphoridae**	Chrysomyia megacephala (Fabricius)	Larvae	[66]
	**Muscidae**	*Musca domestica* L.	Larvae/Adults	[39,63]
	**Tabanidae**	Tabanus mandarinus Schiner	Larvae/Adults	[66]
	**Tachinidae**	*Musca domestica* vicina L.	Larvae/Adults	[65]
	**Tipulidae**	Tipula sp.	Larvae	[28]
**Hemiptera**	**Aetalionidae**	Darthula hardwickii (Gray)	Nymph	[28]
	**Belostomatidae**	Lethocerus indicus Lepeletier and Serville	Adults	[28,66]
	**Cicadidae**	Cicada flammata Distant	Cicada periostracum (exuviae)	[66]
		Cryptotympana atrata (Fabricius)	Adults/Cicada periostracum (Exuviae)	[65,66]
		Cryptotympana mandarina Distant	Cicada periostracum (exuviae)	[66]
		Huechys philamata (Fabricius)	Non-specified	[66]
		Huechys sanguinea (DeGeer)	Non-specified	[66]
		Oncotympana maculaticollis (Motschulsky)	Non-specified	[65]
		Oncotympana maculaticollis (Motschulsky)	Cicada periostracum (exuviae)	[66]
		Platypleura kaempferi (Fabricius)	Non-specified	[65]
		Ericerus pela (Chavannes)	Wax produced by male	[66]
		Notobitus meleagris (Fabricius)	Adults	[28]
	**Dinidoridae**	Aspongopus nepalensis Westwood	Adults	[28]
		Coridius singhalanus Distant	Non-specified	[28]
		Cyclopelta parva Distant	Adults	[66]
	**Fulgoridae**	Lycorma delicatula (White)	Adults	[65,66]
	**Gerridae**	Rhagadotarsus kraepelini Breddin	Non-specified	[66]
	**Nepidae**	Laccotrephes ruber L.	Adults	[28]
	**Pentatomidae**	Aspongopus chinensis Dallas	Adults	[65,66]
		Udonga montana Distant	Adults	[28]
		Nezara viridula smaragdula (Fabricius)	Non-specified	[65]
	**Tessaratomidae**	Tessaratoma papilllosa (Drury)	Adults	[66]
		Tessaratoma quadrata Distant	Adults	[66]
**Hymenoptera**	**Apidae**	Apis andreniformis Smith	Non-specified	[66]
		*Apis cerana* Fabricius	Larvae/Bee venom/Bee wax/Honey/Royal jelly/Bee pollen	[65,66]
		*Apis cerana* indica Fabricius	Larvae/Pupae/Cocoon/Adults/Bee comb/Bee wax/Honey	[28]
		Apis dorsata Fabricius	Larvae/Pupae/Cocoon/Adults/Bee comb/Bee wax/Honey/Pollen	[28,66]
		Apis florea Fabricius	Larvae/Pupae/Cocoon/Honey/Bee comb	[28,66]
		Apis laboriosa Smith	Larvae/Pupae/Cocoon/Honey/Bee comb/Pollen	[28]
		*Apis mellifera* L.	Larvae/Adults/Bee venom/Bee wax/Honey/Royal jelly/Bee pollen/Propolis	[39,63,65,66]
		Cephalotrigona capitata (Smith)	Non-specified	[63]
		Frieseomelitta silvestrii (Friese)	Non-specified	[63]
		Frieseomelitta varia (Lepeletier)	Non-specified	[63]
		Lepidotrigona arcifera (Cockerell)	Honey/Nest	[28]
		Lestrimelitta limao (Smith)	Non-specified	[63]
		Melipona asilvai Moure	Non-specified	[63]
		Melipona compressipes (Fabricius)	Non-specified	[63]
		Melipona mandacaia Smith	Non-specified	[63]
		Melipona marginata Lepeletier	Non-specified	[63]
		Melipona quadrifasciata Lepeletier	Non-specified	[63]
		Melipona scutellaris Latreille	Non-specified	[63]
		Melipona subnitida Ducke	Non-specified	[63]
		Partamona Cupira (Smith)	Non-specified	[63]
		Platynopoda magnifica Cockerell	Non-specified	[66]
		Plebeia emerina (Friese)	Non-specified	[63]
		Tetragonisca angustula (Latreille)	Non-specified	[63]
		Trigona mosquito Smith	Non-specified	[63]
		Trigona spinipes (Fabricius)	Non-specified	[63]
		Xylocopa appendiculata Smith	Adults	[65,66]
		Xylocopa sinensis Smith	Non-specified	[66]
	**Formicidae**	Acromyrmex landolti (Forel)	Non-specified	[63]
		Atta cephalotes L.	Non-specified	[63]
		Atta serdens L.	Non-specified	[63]
		Camponotus japonicus Mayr	Non-specified	[66]
		Camponotus maculatus (Fabricius)	Adults	[39]
		Camponotus sp.	Adults/Nest	[39]
		Campsomeris annulata Fabricius	Non-specified	[66]
		Dinoponera quadriceps Kempf	Non-specified	[63]
		Formica fusca L.	Non-specified	[66]
		Oecophylla smaragdina (Fabricius)	Adults	[28,66]
		Pachycondyla sp.	Nest	[39]
		Polyrhachis dives Smith	Non-specified	[66]
		Polyrhachis vicina Roger	Non-specified	[65,66]
		Solenopsis saevissima (Smith)	Non-specified	[63]
		Tetramorium sp.	Nest	[39]
	**Sphecidae**	Sceliphron sp.	Pupae/Cocoon/Adults/Nest	[39]
	**Vespidae**	Apoica pallens (Fabricius)	Non-specified	[63]
		Brachygastra lecheguana (Latreille)	Non-specified	[63]
		Parapolybia varia (Fabricius)	Adults, Bee comb	[66]
		Polistes canadensis L.	Non-specified	[63]
		Polistes chinensis (Fabricius)	Larvae/Adults/Bee comb	[66]
		Polistes macaensis (Fabricius)	Adults	[65]
		Polybia sericea (Olivier)	Non-specified	[63]
		Protonectarina sylveirae (Saussure)	Non-specified	[63]
		Protopolybia exigua (Saussure)	Non-specified	[63]
		Provespa barthelemyi (Byusson)	Adults	[28]
		Synoeca surinama (L.)	Non-specified	[63]
		Vespa affinis (L.)	Bee comb	[66]
		Vespa bicolor Fabricius	Adults	[65]
		Vespa ducalis Smith	Adults	[65]
		Vespa mandarinia Smith	Bee comb	[28,65,66]
		Vespa nigrithorax Buusson	Bee comb	[66]
		Vespa tropica L.	Larvae/Pupae/Cocoon/Adults/	[28]
		Vespa velutina auraria Smith	Bee comb	[65,66]
		Vespula vulgaris L.	Nest	[39]
**Lepidoptera**	**Bombycidae**	*Bombyx mori* L.	Eggs/Larvae/Pupae/Cocoon/Male adults/*Beauveria bassiana* infected larvae/Pupae/Exuviae	[65,66]
	**Cossidae**	Cossus sp.	Larvae, Adults	[28]
	**Crambidae**	Omphisa fuscidentalis Hampson	Larvae	[28]
	**Erebidae**	Arctia caja (L.)	Non-specified	[66]
	**Hepialidae**	Thitarodes armoricanus Oberthür	*Cordyceps* sp. Infected larvae	[65]
		Erionota torus Evans	Larvae	[28]
	**Lasiocampidae**	Malacosoma sp.	Larvae	[28]
	**Limacodidae**	Cnidocampa flavescens (Walker)	Pupae/Cocoon	[66]
		Monema flavescens Walker	Pupae/cocoon	[65]
		Thosea sinensis Walker	Pupae/Cocoon	[66]
	**Noctuidae**	Agrotis ipsilon (Hufnagel)	*Cordyceps hawkesii* infected larvae	[66]
	**Nymphalidae**	Polygonia *c*-aureum L.	Adults	[65]
	**Papilionidae**	Papilio machaon L.	Larvae/Pupae/Cocoon	[66]
		Papilio xuthus L.	Larvae	[65,66]
	**Pieridae**	Pieris rapae (L.)	Adults	[65,66]
	**Psychidae**	Oiketicus kirbyi Guilding	Non-specified	[63]
	**Pyralidae**	Aglossa dimidiatus Haworth	Frass	[66]
		Ostrinia nubilalis (Hübner)	Larvae	[65,66]
		Proceras venosatum (Walker)	Larvae	[66]
	**Saturniidae**	*Antheraea pernyi* (Guérin-Meneville)	Pupae/Cocoon	[65,66]
		Cirina butyrospermi Vuillot	Non-specified	[39]
		Philosamia cynthia Grote	Larvae/Pupae/Cocoon	[65,66]
		Samia cynthia ricini (Boisduval)	Larvae	[28]
**Mantodea**	**Mantidae**	Hierodula coarctata Saussure	Adults	[28]
		Hierodula patellifera Serville	Eggs/Adults	[65,66]
		Mantis religiosa Linnaeus	Eggs	[66]
		Paratenodera sinensis (Saussure)	Eggs	[66]
		Statilia maculata Thunberg	Eggs/Adults	[65,66]
		Tenodera angustipennis (Saussure)	Eggs	[66]
		Tenodera sinensis Saussure	Egg/Adults	[28,65]
**Neuroptera**	**Myrmeleontidae**	Euroleon sinicus (Navás)	Larvae	[66]
		Myrmeleon sp.	Larvae	[28]
**Odonata**	**Aeschnidae**	Aeschna melanictera Selys	Adults	[66]
		Anax parthenope julius Brauer	Adults	[66]
	**Gomphidae**	Gomphidia confluens Selys	Non-specified	[65]
	**Libellulidae**	Crocothemis servilia Drury	Nymphs	[28,66]
		Diplacodes trivialis Rambur	Nymphs	[28]
		Neurothemis fulvia Drury	Nymphs	[28]
		Orthetrum pruinosum neglectum Rambur	Nymphs	[28]
		Orthetrum sabina Drury	Nymphs	[28]
		Orthetrum triangulare Selys	Nymphs	[28]
		Pantala flavescens Fabricius	Nymphs	[28,65,66]
		Potamarcha congener Rambur	Nymphs	[28]
**Orthoptera**	**Acrididae**	Acrida cinerea (Thunberg)	Adults	[66]
		Acrida cinerea (Thunberg)	Adults	[65,66]
		Ceracris kiangsu Tsai	Non-specified	[66]
		*Locusta migratoria* (L.)	Non-specified	[65,66]
		Melanoplus sp.	Adults	[28]
		Oxya chinensis (Thunberg)	Adults	[65,66]
		Patanga japonica (Bolívar)	Adults	[65,66]
		*Schistocerca gregaria* (Forskål)	Adults	[39]
	**Gryllidae**	*Acheta domesticus* (L.)	Non-specified	[39,63]
		Brachytrupes portentosus (Lichtenstein)	Non-specified	[66]
		Gryllus assimilis (Fabricius)	Non-specified	[63]
		Gryllus mitratus Burmeister	Adults	[66]
		Gryllus sp.	Adults	[28]
		Gryllus testaceus Wallker	Non-specified	[66]
		Loxoblemmus doenitzi Stein	Nymph/Adults	[65,66]
		Scapsipedus micado Saussure	Adults	[65]
		Tarbinskiellus portentosus (Lichtenstein)	Adults	[28]
		Teleogryllus emma (Ohmachi and Matsuura)	Adults	[65]
		Velarifictorus aspersus (Walker)	Adults	[65]
	**Gryllotalpidae**	Gryllotalpa orientalis Burmeister	Nymph/Adults	[65,66]
		Gryllotalpa unispina Saussure	Non-specified	[66]
	**Phalangopsidae**	Paragryllus temulentus Saussure	Non-specified	[63]
	**Tettigoniidae**	Elimaea securigera Brunner von Wattenwyl	Adults	[28]
		Gampsocleis buergeri (De Haan)	Male	[66]
		Gampsocleis gratiosa Brunner Von Wattenwyl	Adults	[65]
		Gampsocleis sedakovii obscura (Walker)	Adults	[65]
		Mecopoda elongata (L.)	Non-specified	[66]
		Pseudophyllus titan White	Adults	[28]
**Phasmatodea**	**Lonchodidae**	Carausius sp.	Adults	[28]
**Psocodea**	**Pediculidae**	Pediculus humanus L.	Non-specified	[63]
**Trichoptera**	**Phryganeidae**	Phryganea japonica McLachlan	Larvae	[66]
**Zygentoma**	**Lepismatidae**	Lepisma saccharina L.	Non-specified	[66]
		Lepisma villosa Fabricius	Non-specified	[66]

Among those 235 species, 151 were documented with the specific stage or product (e.g., feces, nest, etc.) used. Adult stage (90 species) was the most documented stage, followed by larvae/nymphs (60), pupae/cocoon (13), and eggs (7). The usage of adults and larvae/nymph are distributed widely among different orders (e.g., 2/3 orders were documented). On the contrary, the usage of eggs (e.g., Lepidoptera and Mantodea) and pupae/cocoon (e.g., Hymenoptera and Lepidoptera) are limited in two orders, respectively. Besides, fungus infected larvae are only documented in three species in Lepidoptera, which are the *Beauveria bassiana* infected *Bombyx mori* (L.), *Cordyceps sinensis* infected *Thitarodes armoricanus* Oberthür, and *Cordyceps hawkesii* infected *Agrotis ipsilon* (Hufnagel).

Other than insects per se, byproducts from 31 species were documented. Byproducts from species in Hymenoptera are the most documented (e.g., 18 out of 31 species), for example, bee wax, honey, royal jelly, bee pollen, bee comb, and bee venom from the family Apidae, bee comb from the family Vespidae, and nest from the family Formicidae (Table 2).

### 3.2 Health effects of medicinal insects and their associated mechanisms

The international classification of diseases system ICD10 (Table 3) is used here to sort the health effects of insects mentioned in literature except wound healing, which cannot be sorted in a single group of disease. The ICD10 system was published by the World Health Organization (WHO) in 1994 (more details can be found in the ICD10 Interactive Self Learning Tool, https://apps.who.int/classifications/apps/icd/icd10training/). Modern research (i.e., 2012–2022) that studied medicinal functions with species family documented in the above five summarized literatures [28,39,63,65,66] were screened out on Web of Science™. We identified ∼300 articles, which cover 23 families.

**Table 3:** ICD1O code and the associated diseases.

ICD10 code	Disease classified
A00-B99	Certain infectious and parasitic diseases
C00-D49	Neoplasms
D50-D89	Diseases of the blood and blood-forming organs and certain disorders involving the immune mechanism
E00-E89	Endocrine, nutritional, and metabolic diseases
F01–F99	Mental, Behavioral and Neurodevelopmental disorders
G00-G99	Diseases of the nervous system
H00–H59	Diseases of the eye and adnexa
H60–H95	Diseases of the ear and mastoid process
I00–I99	Diseases of the circulatory system
J00-J99	Diseases of the respiratory system
K00–K95	Diseases of the digestive system
L00-L99	Diseases of the skin and subcutaneous tissue
M00-M99	Diseases of the musculoskeletal system and connective tissue
N00–N99	Diseases of the genitourinary system
O00–O99	Pregnancy, childbirth, and the puerperium
P00–P96	Certain conditions originating in the perinatal period
Q00-Q99	Congenital malformations, deformations, and chromosomal abnormalities
R00-R99	Symptoms, signs, and abnormal clinical and laboratory findings, not elsewhere classified
S00-T88	Injury, poisoning and certain other consequences of external causes
U00–U85	Codes for special purposes
V00–Y99	External causes of morbidity
Z00-Z99	Factors influencing health status and contact with health services

**Fig. 3:**
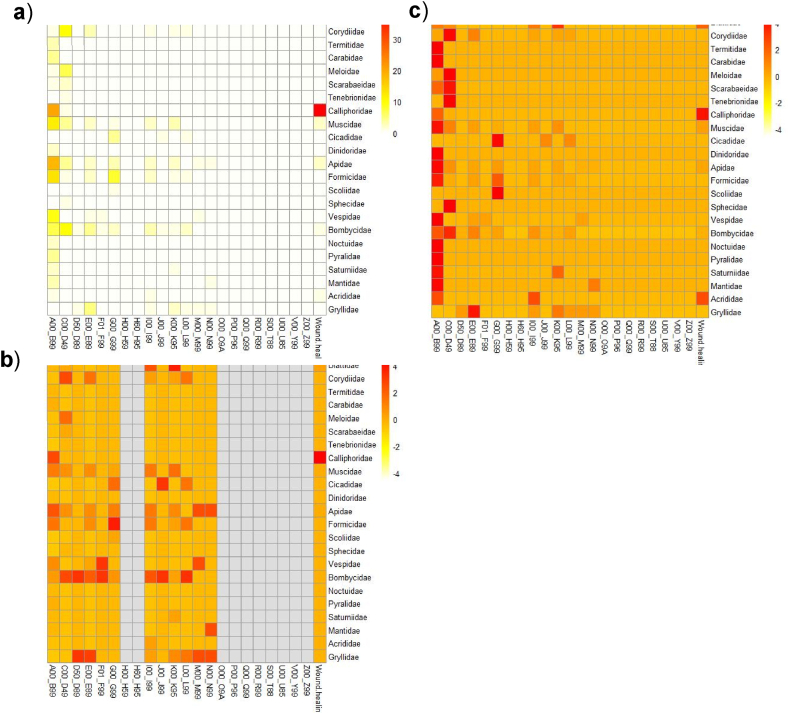
Heatmap of associations between insect families and diseases. **a**) showed with numbers of papers; **b**) scaled by disease; and **c**) scaled by family of insect. International classification of diseases (ICD10) is used here to sort the diseases with code A to Z (https://www.icd10data.com/ICD10CM/Codes). Wound healing is added since it does not belong to any ICD10 code.

The focus on health effects of insects in modern medicine has changed significantly compared to folk medicine. Insects that used to fight against infectious and parasitic diseases (ICD A00-B99) counted ∼36% of the total research, followed by insects promote wound healing (counted ∼17%) and anti-neoplasms (ICD C00-D49, counted ∼15%). Heatmap analysis (Fig. 3a–c) showed the association between diseases and the insect families. Insect families used in different groups of diseases are diverse except wound healing, which was heavily focused on the use of Calliphoridae (Fig. 3b). For example, infection related diseases were frequently used with insects in Calliphoridae, Muscidae, Apidae, and Formicidae. Neoplasm studies frequently used insects in Corydiidae, Meloidae, and Bombycidae. Distributions of associated disease in each insect family vary (Fig. 3c). For example, Blattidae was mostly used in digestive system disease research. Cicadidae and Scollidae were mostly used in nervous system disease research.

The choice of insects in disease research seems to be heavily impacted by the documentary of folk medicine and the market availability. Below is a detailed description of functions mentioned in modern research based on the ICD system with mechanisms (Table 4) and ingredients (Table 5) documented.

**Table 4:** Health effects of medicinal insects and the associated mechanisms documented.

ICD 10	Effects	Mechanisms	Reference(s)
A00-B99	Antibacteria	Peptide deformylase	[67]
		Membrane permeability alteration and disruption	[68]
		DNA formation inhibition and damage	[68]
		Biofilm damage	[69]
	Antifungus	Reduced bacterial adherence to human keratinocytes	[70]
		Membrane disruption	[71]
	Antivirus	ROS production	[72]
	Parasite inhibition	Potentiating innate immunity function	[73]
C00-D49	Antitumor	Inhibit cell adhesion	[72]
		Restrain cell migration and invasion	[72]
		Antiproliferation	[74]
		Apoptosis	[75]
		Immunomodulatory	[74]
		Anti-oxidation	[76]
		Reduce inflammation	[76]
E00-E89	Antihyperglycemic and antidiabetic	Advanced glycation end products (AGEs) inhibition	[77,78]
		α-glucosidase inhibition	[79,80]
		Beta cell function improvement	[81]
	Reduce blood lipid and prevent obesity	Energy metabolism balance regulation	[82]
		AMPK/mTOR signaling pathway activation	[82]
		Cholesterol metabolism-related biochemical parameters regulation	[83]
	Combating malnutrition	Protein supplement	[84]
F01–F99	Anti-anxiety	may work with the B2-receptors and B1-receptors	[85]
	Anti-depression	antioxidant and estrogenic properties	[86]
G00-G99	Neuroprotection	Anti-oxidation	[87]
		Cell cycle inhibition	
		preventing cyclin D1 up-regulation	[87]
		Reduce inflammation	
		Mitogen activated protein kinase (MAPK) inhibition	[87]
		Prevent apoptosis	
		Bax inhibition	[88]
		Caspase-3 inhibition	[88,89]
		Nrf2/HO-1 pathway regulation	[90,91]
		BDNF/TrkB pathway regulation	[90]
		Nurr1 expression	[92]
	Venom immunotherapy	Specific IgE reduction and IgG4 induction	[93]
I00–I99	Against thrombosis	FXa inhibition	[94]
		Antiplatelet aggregation	[94,95]
		Plasminogen activation and fibrin (ogen) hydrolyzation	[80]
	Against hypertension	Regulating vascular tone	[96]
		Angiotensin-converting enzyme (ACE) inhibition	[96,97]
J00-J99	Anti-tussive and anti-asthma	Cytokines and neuropeptides regulation	[98]
		TRPA1/TRPV1/TRPV5 channels regulation	[98]
		GATA-3/Th2 and IL-17/RORγt pathways regulation	[99]
K00–K95	Hepatoprotection	Reduce inflammation	[100,101]
		Relevant signaling pathways regulation	[101]
		Anti-oxidation	[100]
	Gastroprotection	Reduce inflammation	[102,103]
		NF-kappa B signaling pathway regulation	[104,105]
		Anti-oxidation	[103]
		Intestinal microbiota regulation	[[106], [107], [108], [109]]
		Neovascularization	[102]
		Growth factor expression enhancement	[102]
L00-L99	Reduce melanogenesis	NA	[110]
	Photoaging protection	Reduced UVB-induced skin winkles	[111]
		Anti-oxidation	[111,112]
		Reduce inflammation	[111]
		Alleviated the epidermal barrier dysfunction	[111]
		Reduce collagen breakdown	[111]
	Psoriasis	Reduced immune response	[113]
		Attenuated epidermal proliferation	[113]
	Dermatitis	Reduce inflammation	[2]
Wound healing	Wound healing	Biosurgical debridement	[114]
		Disinfection	
		Anti-bacteria	[[115], [116], [117]]
		Wound healing	
		Stimulated keratinocytes	[118,119]
		Pro-fibrogenic and pro-angiogeneic effects	[120,121]
		Blood coagulation	[114]
		Cell proliferation, tissue reconstruction	[104,115,122]
		Reduce inflammatory cytokines	[115,123,124]
		Glycosidases (glycoside hydrolases)	[124]

**Table 5:** Medicinal insect species and their effective ingredients.

ICD 10	Order	Family	Species	Ingredients	Reference(s)
A00-B99	Blattodea	Blattidae	*Periplaneta americana* L.	Unsaturated fatty acid	[125]
				Gut microbiota	[126]
		Termitidae	*Odontotermes formosanus* (Shiraki)	Microbiota	
			*Macrotermes* sp.	Actinomycetes	[127]
	Coleoptera	Meloidae	Meloidae sp.	Terpenoid - Cantharidin	[128]
		Scarabaeidae	*Copris tripartitus* Waterhouse	AMP - Coprisin	[129]
	Diptera	Muscidae	*Musca domestica* L.	Proteins - Lectin	[130]
				AMP - Cecropin, attacin, lebocin	[131]
		Calliphoridae	*Lucilia sericata* (Meigen)	AMP - Lucifensin, lucimycin, attacins, cecropins, diptericins, proline-rich peptides, and sarcotoxins	[132]
			*Cochliomyia macellaria* (Fabricius)	Excretions and secretions	[133]
			*Calliphora Vicina* Robineau-Desvoidy	Excretions and secretions	[134]
			*Sarconesiopsis magellanica* (Le Guillou)	Excretions and secretions	[135]
		Drosophilidae	*Drosophila melanogaster* Meigen	AMP – Drosocin, Mtk-1, Mtk-2	[136]
	Hemiptera	Dinidoridae	*Coridius chinensis* (Dallas)	lysozyme - CcLys2	[137]
				AMP - CcAMP1	[138]
	Hymenoptera	Apidae	*Melipona scutellaris* Latreille	AMP - meliponamycin A, meliponamycin B	[139]
			*Melipona orbignyi* (Guérin-Méneville)	Geopropolis extract	[140]
		Formicidae	*Apterostigma dentigerum* Wheeler	Microbiota – *Pseudonocardia* producing antibiotic (pseudonocardones)	[141]
			*Tetramorium bicarinatum* (Nylander)	AMP - Bicarinalin	[142]
	Hymenoptera	Vespidae	*Agelaia pallipes* (Olivier)	AMP - pronectin	[143]
			*Polybia dimorpha* Richards	AMP - Polydim-I	[144]
			*Polybia paulista* Ihering	AMP - Polybia-CP	[72]
			*Vespa affinis* L.	AMP - Mastoparan-AF	[145]
	Lepidoptera	Bombycidae	*Bombyx mori* L.	AMP - Cecropin A, Cecropin B, moricin	[146]
				Microbiota - Yeast-melanin	[147]
		Noctuidae	*Spodoptera litura* (Fabricius)	AMP - Lebocin	[148]
	Mantodea	Mantidae	Mantidis sp.	Ootheca lipid extract - Sesquiterpenoids, monoterpenes	[149]
			*Sphodromantis viridis* Forsskål	AMP - Mastoparan-S	[150]
C00-D49	Blattodea	Corydiidae	*Eupolyphaga sinensis* (Walker)	Protein - EPS72	[125]
				Polysaccharide	[74]
	Coleoptera	Meloidae	*Mylabris* sp.	Norcantharidin	[81,151]
			*Mylabris phalerata* Pallas	Cantharidin	[15,152,153]
		Scarabaeidae	*Copris tripartitus* Waterhouse	Coprisin - CopA3	[154]
			*Scarabaeus sacer* L.	Chitosan	[155]
	Diptera	Calliphoridae	*Chrysomya albiceps* (Wiedemann)	Carboxymethyl derivative of chitosan	[156]
			*Sarcophaga aegyptiaca* (Salem)	Carboxymethyl derivative of chitosan	[156]
		Muscidae	*Musca domestica* L.	Anti-tumor peptide	[157,158]
				Microbiota - *Bacillus subtilis* - extracellular polymeric substance	[159]
	Lepidoptera	Bombycidae	*Bombyx mori* L.	*Beauveria bassiana* infected larvae	
				Cyclodepsipeptide - bassianolide	[160]
				Cordycepin	[161]
				Cecropin A	[146]
				Peptide - BmCecA and BmCecD	[162]
	Hymenoptera	Streptomycetaceae	*Sceliphron madraspatanum* (Fabricius)	Micorbiota - *Streptomyces* sp. - strepantibins A-C	[163]
E00-E89	Lepidoptera	Bombycidae	*Bombyx mori* L.	Flavonoids and free amino acids	[80]
	Blattodea	Corydiidae	*Eupolyphaga sinensis* (Walker)	Peptide DP17	[82]
				Peptide (AR-9)	[164]
	Diptera	Muscidae	*Musca domestica* L.	Extract	[79,83]
	Hymenoptera	Apidae	*Apis mellifera* L.	Propolis - epicatechin and *p*-coumaric	[165]
			*Bombus ignitus* (Smith)	Glycosaminoglycan	[166]
		Vespidae	*Vespa basalis* Smith	Peptide- Mastoparan B	[167]
	Orthoptera	Gryllidae	*Gryllus bimaculatus* De Geer	Glycosaminoglycan	[166,168]
				Ethanol extract	[169]
			*Gryllus assimilis* (Fabricius)	Protein hydrolysates	[77]
F01-f99	Hymenoptera	Vespidae	*Polybia paulista* Ihering	venom	[85]
	Lepidoptera	Bombycidae	*Bombyx mori* L.	Silk syrup	[86]
G00-G99	Diptera	Muscidae	*Musca domestica* L.	Larval meal	[87]
	Hemiptera	Cicadidae	*Cryptotympana pustulata* Fabricius	Cicadidae periostracum - *N*-acetyldopamine dimers	[170]
			Cicadidae sp.	Cicadidae periostracum - cyclic peptide	[89]
	Hymenoptera	Apidae	*Apis mellifera* L.	Venom - melittin	[88]
			Apidae sp.	Propolis - Caffeic acid phenethyl ester	[90]
		Formicidae	*Polyrhachis dives* Smith	Dopamine derivatives	[61]
			*Myrmecia pilosula* F. Smith	Venom	[171]
			*Dinoponera quadriceps* Kempf	Venom	[[172], [173], [174]]
		Scoliidae	*Scolia decorata ventralis* Smith	Venom - peptides	[175]
I00–I99	Blattodea	Corydiidae	*Eupolyphaga sinensis* Walker	Serine proteases	[176]
				Protein	[176]
		Blattidae	*American cockroach* L.	Xinmailong	[96]
	Hymenoptera	Apidae	*Apis mellifera* L.	Polyphenol - epicatechin and *p*-coumaric	[165]
		Formicidae	*Dinoponera quadriceps* Kempf	Venom	[95]
			*Oecophylla smaragdina* Fabricius	Proteins	[97]
	Lepidoptera	Bombycidae	*Bombyx mori* L.	Protein - sericin	[177]
				Peptide	[178]
				Pupae oil	[179]
	Orthoptera	Acrididae	*Oxya chinensis sinuosa* Mistshenko	*N*-acetyldopamine dimers	[94]
		Gryllidae	*Gryllus assimilis* (Fabricius)	Protein hydrolysates	[77]
K00–K95	Blattodea	Blattidae	*Periplaneta americana* L.	Extracts	[101,107]
				Oligosaccharides	[106])
				Ethanol extract - Kangfuxin	[102,105]
				Antimicrobial peptide (Periplanetasin-2)	[103]
		Corydiidae	*Eupolyphaga sinensis* (Walker)	Peptide	[109]
	Diptera	Muscidae	*Musca domestica* L.	Low molecular weight peptides	[108,180]
			*Stomoxys calcitrans* L.	Metabolites	[181]
	Hymenoptera	Apidae	*Trigona* sp.	Honey	[182]
		Formicidae	*Oecophylla smaradina* Fabricius	Ethanolic extract	[183] (2019)
	Lepidoptera	Saturniidae	*Antheraea pernyi* (Guérin-Méneville)	Silk fibroin	[184]
		Bombycidae	*Bombyx mori* L.	Peptide - Gloverin A2 (BMGlvA2)	[185]
	Orthoptera	Crididae	*Oxya chinensis sinuosa* (Mistshenko)	Extracts	[186]
		Gryllidae	*Gryllus bimaculatus* De Geer	Extracts	[100,111]
			*Protaetia brevitarsis* (Lewis)	Extracts	[186]
L00-L99	Blattodea	Corydiidae	*Eupolyphaga sinensis* (walker)	Polypeptides	[112]
	Coleoptera	Scarabaeidae	*Allomyrina dichotoma* L.	Extract	[111]
			*Protaetia brevitarsis seulensis* (Kolbe)	Extract	[111]
		Tenebrionidae	*Tenebrio molitor* L.	Extract	[111]
	Hemiptera	Cicadidae	Cicadidae sp.	Cicadidae Periostracum	[2]
	Lepidoptera	Bombycidae	*Bombyx mori* (L.)	Freeze-dried mature silkworm powder	[110]
				Cocoon sericin	[187]
				Feces	[2]
	Orthoptera	Gryllidae	*Gryllus bimaculatus* De Geer	Extract	[111]
Wound healing	Blattodea	Blattidae	*Periplaneta americana* (L.)	Extracts	[122]
				Phenolic Derivatives	[188]
				Periplanpyrazine	[189]
				Kangfuxin liquid	[120]
	Diptera	Calliphoridae	*Lucilia sericata* (Meigen)	Excretions/secretions	[124,190,191]
				DNAse	[192]
				Angiopoietin-1 enzyme	[193]
				Allantoin	[194]
				Lysozymes	[195]
				Signal peptide protease	[196]
				Prenyl metalloproteinase	[196]
				Serine protease	[114,196,197]
				Chymotrypsin	[198]
			*Sarconesiopsis magellanica* (Le Guillou)	Excretions/secretions	[199]
				AMP	[68,200]
				Proteases	[201]
				Fat body and hemolymph extract	[121]
	Hymenoptera	Apidae	*Apis mellifera* L.	Venom	[118]
	Lepidoptera	Bombycidae	*Bombyx mori* (L.)	Silk fibroin	[185]

#### 3.2.1 Health effects associated with infectious and parasitic diseases (A00-B99)

Most (e.g., ∼65%) of the research focused on antibacterial effects. At least 30 species of bacteria were determined to be inhibited by insect derivatives (Table 6), including but not limiting to bacteria associated with wound infection (e.g., *Bacillus* sp., *Staphylococcus* sp., and *Proteus* sp.), digestive system infection (e.g., *Helicobacter pylori*, *Bacillus cereus*, *Citrobacter freundii*, *Escherichia coli*, and *Salmonella enterica*), urinary tract infection (e.g., *Enterobacter cloacae*, *Enterococcus faecalis*, *Acinetobacter baumannii*, and *Serratia marcescens*), and other infections (e.g., *Listeria monocytogenes* and *Haemophilus influenzae*). Besides, at least 13 species of fungus (*Aspergillus* sp., *Penicillium* sp., *Trichoderma* sp., and *Candida* sp.) (Table 7), five viruses (e.g., Rift Valley fever virus, Coxsackie B4 virus, Hepatitis B virus, Hepatitis A virus, and Herpes simplex virus) (Table 8), and ten parasites (e.g., *Trypanosoma cruzi*, *Leishmania* sp. *Plasmodium* sp., and *Haemonchus contortus*) (Table 9) were determined can be inhibited by insect derivatives. Mechanisms include reducing bacterial adherence to human keratinocytes [70], biofilm interruption [69], membrane permeability alteration and disruption [68], peptide deformation [67], ROS production [72], and DNA formation inhibition and damage [68]. The active ingredients are mainly antimicrobe peptides (AMP). For example, coprisin [129], lebocin [148], drosocin [136], pronectin [143], cecropin [146], etc. Besides, certain unsaturated fatty acid [125], protein (e.g., lectin [130] and lysozyme [137], and terpenoid (e.g., cantharidin [128]) also showed antimicrobe/virus effects. The insect associated microbes contributed as well, for example the actinomycetes isolated from *Termitidae* sp [127]. and the melanin extracted from yeast in *B. mori* [147].

**Table 6:** Bacteria inhibited by medicinal insects with insect species shown as examples.

Bacteria Inhibited	Order	Family	Species	Stage or ingredients used	Reference(s)
*Helicobacter pylori*	Propolis from bees with no specific species mentioned			Propolis	[67]
Micrococcus flavus	Coleoptera	Carabidae	Calosoma sycophanta L.	Secretions	[202]
Micrococcus luteus	Lepidoptera	Bombycidae	*Bombyx mori* (L.)	Antimicrobial peptides	[203]
Micrococcus tetragenus	Blattodea	Termitidae	Odontotermes formosanus (Shiraki)	Associated microbiota	[127]
Mycobacterium abscessus subsp. Massiliense	Hymenoptera	Vespidae	Polybia dimorpha Richards	Venom	[144]
Bacillus pumilus	Lepidoptera	Saturniidae	Antheraea mylitta (L.)	Antimicrobial peptides	[204]
Bacillus subtilis	Lepidoptera	Bombycidae	*Bombyx mori* (L.)	Ethyl acetate extract	[203]
Bacillus cereus	Coleoptera	Carabidae	Calosoma sycophanta L.	Secretions	[202]
Listeria monocytogenes	Coleoptera	Carabidae	Calosoma sycophanta L.	Secretions	[202]
Methicillin-resistant *Staphylococcus aureus*	Blattodea	Blattidae	*Periplaneta americana* (L.)	Associated microbiota	[205]
*Staphylococcus aureus*	honeybee-specific lactic acid bacteria with no specifc species mentioned			Associated microbiota	[206]
Staphylococcus epidermidis	Diptera	Calliphoridae	*Lucilia cuprina* (Wiedemann)	Secretions	[207]
Staphylococcus xylosus	Hymenoptera	Formicidae	Tetramorium bicarinatum (Nylander)	Antimicrobial peptides	[142]
Streptococcus pyogenes	Hymenoptera	Apidae	Frieseomelitta nigra (Cresson)	Honey	[208]
Citrobacter freundii	honeys with no specific species mentioned			Honey	[209]
*Enterobacter cloacae*	Blattodea	Blattidae	*Periplaneta americana* (L.)	Associated microbiota	[126]
*Enterococcus faecalis*	Diptera	Calliphoridae	*Lucilia sericata* (Meigen)	Antimicrobial peptides	[132]
*Escherichia coli*	Hymenoptera	Apidae	Melipona orbignyi (Guérin-Méneville)	Geopropolis	[140]
Klebsiella pneumonia	honeys with no specific species mentioned			Honey	[209]
*Proteus mirabilis*	Diptera	Calliphoridae	*Lucilia sericata* (Meigen)	Secretions	[69]
Proteus vulgaris	Diptera	Calliphoridae	*Lucilia sericata* (Meigen)	Antimicrobial peptides	[132]
*Salmonella enterica*	Blattodea	Blattidae	*Periplaneta americana* (L.)	Associated microbiota	[126]
Salmonella infantis	honeys with no specific species mentioned			Honey	[209]
*Salmonella typhimurium*	Coleoptera	Carabidae	Calosoma sycophanta L.	Secretions	[202]
Legionella gormanii	Lepidoptera	Pyralidae	Galleria mellonella (L.)	Hemolymph polypeptides	[210]
Acinetobacter baumannii	honeys with no specific species mentioned			Honey	[209]
Haemophilus influenzae	Hymenoptera	Apidae	Frieseomelitta nigra (Cresson)	Honey	[208]
*Pseudomonas aeruginosa*	Diptera	Calliphoridae	*Lucilia cuprina* (Wiedemann)	Secretions	[207]
Pseudomonas fluorescens	Hymenoptera	Formicidae	*Solenopsis invicta* (Buren)	Venom	[211]
*Serratia marcescens*	Diptera	Calliphoridae	Chrysomya sp.	Secretions	[212]

**Table 7:** Fungus inhibited by medicinal insects with insect species shown as examples.

Fungus inhibited	Order	Family	Species	Stage or ingredients used	References
*Aspergillus flavus*	Blattodea	Blattidae	*Periplaneta americana* (L.)	Associated microbiota	[126]
*Aspergillus fumigatus*	Blattodea	Blattidae	*Periplaneta americana* (L.)	Associated microbiota	[126]
*Aspergillus niger*	Blattodea	Blattidae	*Periplaneta americana* (L.)	Associated microbiota	[126]
*Aspergillus ochraceus*	Coleoptera	Carabidae	*Calosoma sycophanta* L.	Secretions	[202]
*Aspergillus versicolor*	Coleoptera	Carabidae	*Calosoma sycophanta* L.	Secretions	[202]
*Aspergillus flavus*	Blattodea	Blattidae	*Periplaneta americana* (L.)	Associated microbiota	[126]
*Penicillium funiculosum*	Coleoptera	Carabidae	*Calosoma sycophanta* L.	Secretions	[202]
*Penicillium italicum*	Blattodea	Blattidae	*Periplaneta americana* (L.)	Associated microbiota	[126]
*Penicillium ochrochloron*	Coleoptera	Carabidae	*Calosoma sycophanta* L.	Secretions	[202]
*Penicillium verrucosum* var*. Cyclopium*	Coleoptera	Carabidae	*Calosoma sycophanta* L.	Secretions	[202]
*Trichoderma viride*	Coleoptera	Carabidae	*Calosoma sycophanta* L.	Secretions	[202]
*Trichophyton rubrum*	*Blattodea*	Termitidae	*Nasutitermes* sp.	Associated microbiota	[213]
*Candida albicans*	Blattodea	Blattidae	*Periplaneta americana* (L.)	Associated microbiota	[126]

**Table 8:** Virus inhibited by medicinal insects with insect species shown as examples.

Virus inhibited	Order	Family	Species	Stage or ingredients used	References
Rift Valley Fever virus	Diptera	Calliphoridae	*Lucilia cuprina* (Wiedemann)	Secretions	[214]
Coxsackie B4 virus	Diptera	Calliphoridae	*Lucilia cuprina* (Wiedemann)	Secretions	[214]
Hepatitis B virus	Blattodea	Corydiidae	*Eupolyphaga sinensis* (Walker)	Polysaccharide	[73]
Hepatitis A virus	Coleoptera	Curculionidae	*Rhynchophorus ferrugineus* (Olivier)	Larval extract	[215]
Herpes simplex virus	Coleoptera	Curculionidae	*Rhynchophorus ferrugineus* (Olivier)	Larval extract	[215]

**Table 9:** Parasites inhibited by medicinal insects with insect species shown as examples.

Parasite inhibited	Order	Family	Species	Stage or ingredients used	References
Trypanosoma cruzi	Hymenoptera	Formicidae	*Dinoponera quadriceps* (Kempf)	Dinoponeratoxin peptides	[216]
Leishmania infantum	Hymenoptera	Apidae	*Melipona scutellaris* Latreille	Associated microbiota	[139]
Leishmania panamensis	Diptera	Calliphoridae	*Lucilia sericata* (Meigen)	Secretions	[135]
Leishmania major	Diptera	Calliphoridae	*Lucilia sericata* (Meigen)	Secretions	[217]
Leishmania amazonensis	Diptera	Muscidae	*Musca domestica* (L.)	Larvae	[218]
Leishmania tropica	Diptera	Calliphoridae	*Lucilia sericata* (Meigen)	Secretions	[219]
Leishmania donovani	Hymenoptera	Formicidae	*Cyphomyrmex* sp.	Associated microbiota	[220]
Plasmodium falciparum	Diptera	Drosophilidae	*Drosophila melanogaster* Meigen	Antimicrobial peptides	[136]
Plasmodium berghei	Hymenoptera	Formicidae	*Apterostigma dentigerum* Wheeler	Associated microbiota	[141]
Haemonchus contortus	Hymenoptera	Formicidae	*Neoponera* sp.	Venoms	[221]

#### 3.2.2 Health effects associated with neoplasms (C00-D49)

At least 15 types of anticancer activities were documented, including breast cancer, liver cancer, colorectal cancer, lung cancer, ovarian cancer, colon cancer, pancreatic cancer, esophageal cancer, cervical cancer, tongue cancer, bladder cancer, leukemia, murine melanoma, and two types of ascites cancer. Insect-derived ingredients can inhibit tumor cell adhesion [222], restrain cell migration and invasion [222], and induce cell antiproliferation [223] and apoptosis [146] by regulating different pathways, for example the Akt [75], Mapk [224], and PKC [91] pathways. In addition, the antioxidant [225], anti-inflammatory [76], and immunomodulatory [74] functions of insect-derived ingredients contribute to their antitumor effects. Cantharidin from blister beetles (Meloidae) [153], cordycepin [161], and cecropin [146] from silkworms (Bombycidae) have gain many attentions with their anti-tumor effects. Since the cantharidin has certain toxic side effects, a synthetic derivation of cantharidin named noncantharidin has been developed and widely used in modern anti-tumor medicine [81]. *Eupolyphaga sinensis* (Walker) (Blattodea: Corydiidae) is another well-known traditional Chinese anti-tumor medicine, from which the extracted polysaccharide [74] and a protein named EPS72 [125] have been determined as the active ingredients. Recently, chitosan derivations from scarab beetles (Scarabaeidae) [155] and blowflies (Calliphoridae) [156] were determined to be the effective ingredients as well.

#### 3.2.3 Health effects associated with blood and blood-forming organs and certain disorders involving the immune mechanism (D50-D89)

Not much research can be classified in this group besides nutritional anemia. Insects are rich in nutrients and have been proved to be effective diet supplements [226]. For example, the consumption of cricket could help to prevent children nutritional anemia by providing sufficient energy, iron, and zinc [227].

#### 3.2.4 Anti-hyperglycemia and anti-hyperlipidemia are the two major effects associated with endocrine, nutritional, and metabolic diseases (E00-E89)

The anti-hyperglycemic effect works through advanced glycation end products (AGEs) inhibition [78], α-glucosidase inhibition [176], and beta cell improvement [79]. Active ingredients, for example flavonoids and free amino acids from *B. mori* [80] and epicatechin and *p*-coumaric from *A. mellifera* propolis [165] have shown the ability to regulate blood sugar and prevent/treat diabetes. *Anti*-hyperlipidemic effects works through energy metabolism balancing [82], AMPK/mTOR pathway activation [82], and cholesterol metabolism-related biochemical parameters regulation [83], and therefore showed obesity prevention potentials [167]. Peptides, for example, DP17 [82] and AR-9 isolated from *E. sinensis* [164] and Mastoparan B isolated from *Vespa basalis* Smith (Hymenoptera: Vespidae) [167] and glycosaminoglycan from *Gryllus bimaculatus* De Geer (Orthoptera: Gryllidae) [166] and *Bombus ignitus* (Smith) (Hymenoptera: Apidae) [166] have been determined to be the effective ingredients.

#### 3.2.5 Anti-anxiety and anti-depression were the documented effects that associated with mental, behavioral, and neurodevelopmental disorders (F01–F99)

The bradykinin-related peptide isolated from *Polybia paulista* Ihering (Hymenoptera: Vespidae) venom was determined to be the active ingredients again anxiety, which may work with the B2-receptors and B1-receptors [85]. The silk syrup produced from *B. mori* cocoon was determined to have anti-depression effect, which may be due to its antioxidant and estrogenic properties [86].

#### 3.2.6 Neuroprotection and venom immunotherapy are the major functions associated with nervous system (G00-G99)

The mechanisms of insect neuroprotective effects include antioxidation [87], anti-inflammation [87], cell cycle inhibition [87], and apoptosis prevention [88], which can prevent neurodegenerative diseases (e.g., Alzheimer's disease) [87], Parkinson's disease [170], Amyotrophic lateral sclerosis [228], and epilepsy [174]. Venom immunotherapy is an effective treatment for systemic allergic reactions to Hymenoptera venom. The potential mechanisms (e.g., the initial desensitization of effector cells, the regulation of IgG and IgE level, and the associated inflammatory effects) were recently reviewed by Demšar Luzar et al. [229]. Hymenoptera venom was widely documented as traditional medicine or therapy targeting the nervous system and has been studied and used in the modern medicinal system. Peptide [175] and melittin [88] isolated from venom are documented as the effective ingredients.

#### 3.2.7 Anti-thrombosis and anti-hypertension are the mostly documented effects associated with circulatory system (I00–I99)

Anti-thrombosis works through plasminogen activation and fibrinogen (a major determinant of plasma and blood viscosity) hydrolyzation [80], FXa inhibition, and antiplatelet aggregation [94]. Anti-hypertensive effect mainly works through angiotensin-converting enzyme (ACE) inhibition [178]. The identified effective ingredients are, for example, serine proteases from *E. sinensis* [176], sericin from *B. mori* [187], polyphenol (e.g., epicatechin and *p*-coumaric) from *A. mellifera*, and *N*-acetyldopamine dimers from *Oxya chinensis sinuosa* Mishchenko (Orthoptera: Acrididae) [94].

#### 3.2.8 Anti-tussive and anti-asthmatic effects are health functions associated with respiratory system (J00-J99)

*Bombyx batryticatus* (i.e., the dried silkworm larvae after infected by fungi *Beauveria bassiana*) and cicada periostracum (i.e., the cast-off shell of the cicada *Cryptotympana pustulata* (Fabricius)) were well known against respiratory disease in traditional Chinese medicine and have been recommended as potential medicines fighting against SARS-CoV-2 [230]. They help against respiratory disease through cytokines and neuropeptides [98], TRPA1/TRPV1/TRPV5 channels [98], and GATA-3/Th2 and IL-17/RORγt pathways [99] regulations.

#### 3.2.9 Hepatoprotection and gastroprotection are key effects associated with digestive system (K00–K95)

The inflammation reduction [101] and anti-oxidation [100] effects of insects play important role in against the digestive system disease such as diarrhea [181], gastric ulcer [102], and prevent liver damage after acute alcohol exposure [101]. Intestinal microbiota regulation is another key mechanism for gastroprotection [106,107]. Besides, neovascularization and growth factor expression enhancement were determined in preventing recurrence of gastric ulcer [102]. The gastroprotective effect of peptides isolated from *B. mori* [185], *E. sinensis* [109], *Musca domestica* L. (Diptera: Muscidae) [180], and *P. americana* [103] have been confirmed. Among the species tested, the *P. americana* gained a lot of attention in digestive system protection, from which the oligosaccharides [106] and an antimicrobial peptide (Periplanetasin-2) [103] have been identified as effective ingredients. The extract of *P. americana* has been developed into a commercial medicine named Kangfuxin solution in China [102].

#### 3.2.10 Health effects associated with skin and subcutaneous tissue (L00-L99)

Besides the antibacterial function described in group A00-B99, the antioxidation and anti-inflammation effects of insects help to reduce psoriasis [177], dermatitis [2] and UVB-induced melanogenesis [110] and aging [112]. For example, silkworms *B. mori* have been used in skin protection for a long history. The cocoon sericin [187], freeze-dried silkworm powder [110], and even the feces [2] were determined contributed to skin protection.

#### 3.2.11 Health effects associated with musculoskeletal system and connective tissue (M00-M99)

Insects used in rheumatism and arthritis are well-known in traditional medical systems. The anti-inflammation [231] and anti-oxidation [232] effects of insects confirmed in modern research revealed the mechanisms underline. Glycosaminoglycan extracted from *G. bimaculatus* was determined to be an effective ingredient, which produced a significant anti-edema effect [231].

#### 3.2.12 Health effects associated with wound healing

Wound healing is one of the research hot spots in medicinal insects, which does not belong to any ICD 10 categories since many diseases can lead to wound formation. Wound healing can be separated into therapy with and without maggots. Debridement (i.e., the process of larval feeding on necrotic tissues), disinfection (i.e., anti-bacteria functions mentioned above) and wound healing (e.g., through keratinocytes stimulation [119], cell proliferation [122], blood coagulation [114], and pro-angiogenesis [122]) are the three main mechanisms. *Lucilia sericata* (Meigen) is the most used insect in maggot therapy. The excretions and secretions from maggot larvae have shown outstanding effects in wound healing, from which mainly proteins/enzymes (e.g., angiopoietin-1 enzyme [193] and serine protease [197] were determined effectively promote angiogenesis and cell proliferation. Besides, insect-derived products such as honey, bee venom, chitosan, and sericin have used in wound healing [233].

## 4 Medicinal uses of common edible insects

Most forms of traditional medicine rely on plants and plant-derived components [26]. Nevertheless, for centuries, animals are often used as part of folk pharmacopoeia [28,234,235]. Both domesticated and wild fauna resources are used in zootherapy, which involves the application of animals to treat diseases and include them in magic rituals and religious rites [236]. Medicines originating from animals are made either directly from the whole animal or its parts [235]. Insects in medicine fall under an umbrella terminology called “integrative medicine”, which refers to a medical practice that blends traditional treatment with complementary and alternative medicine techniques and, has been safe and effective through scientific research [[237], [238], [239], [240]]. Edible insects are rich in proteins, fats, fiber, vitamins, and minerals but also seem to contain large amounts of polyphenols able to have a key role in specific bioactivities as antioxidant functions. They also exert other activities, such as anti-inflammatory and anticancer activity, antityrosinase, antigenotoxic, and pancreatic lipase inhibitory activities.

Because of bee's medicinal and nutritional benefits, honey has been utilized for thousands of years [241]. In many societies, honey, a bee product, has long been regarded as a therapeutic remedy, and there are about 300 types of honey worldwide [242]. Honey, bee pollen, propolis, royal jelly, beeswax, and even bee venom are some honeybee products that have been used in folk medicine for millennia across the globe [243]. Anti-inflammatory, antimicrobial, antifungal, antiviral, and antioxidant properties have all been observed in these insect-derived products. These antioxidant, antimicrobial, and other medicinal properties are more effective than sucrose in treating diabetes [242]. Another important product from bees, bee venom, has been utilized as a treatment method in East Asia since the second century, making it one of the region's oldest medical practicesf [244]. The chemical structure of bee venom is intricate, involving many different enzymes, peptides, proteins, smaller molecules (amino acids, catecholamines, carbohydrates, and minerals), and lipids that make up honeybee venom [245]. It also contains Melittin, apamin, MCD peptide, histamine, hyaluronidase, and phospholipase-A2 for bee venom's primary components. However, melitin, a peptide obtained from the European honeybee *Apis mellifera* has been well-studied by several authors [246]. Due to its high cytolytic action, it has proven to be highly effective against tumours [247,248]. Bee venom is an allergen agent that causes Asthma, allergic rhinoconjunctivitis, and atopic eczema by stimulating the production of allergen specific CD4^+^ T cells in susceptible individuals [245]. Bees provide health benefits because they contain many different metabolites, such as folic acid, thiamine, biotin, niacin, tocopherol, polyphenols, phytosterols, and enzymes and coenzymes. The beneficial properties include antioxidant, antibacterial, antifungal, and hepatoprotective [241,249]. A recent study by Amr et al. [250] on female rats showed that boneybee products had the potential to reduce oxidative stress, increase cadmium (Cd) excretion via the kidneys, and modify intestinal absorption of the metal.

The larvae of the Australian Sawfly contain novel macrocarpa and grandinol. These chemical components were assessed against *Bacillus subtilis* and showed positive antimicrobial effects against the bacteria [6,251]. The larvae of Sawfly *Tenthredo zonula* Klug also contain phenolic compounds, such as flavonoid glycosides, flavonol oligoglycosides, and naphthodianthrones, and have been evaluated for their health properties [252].

**Fig. 4:**
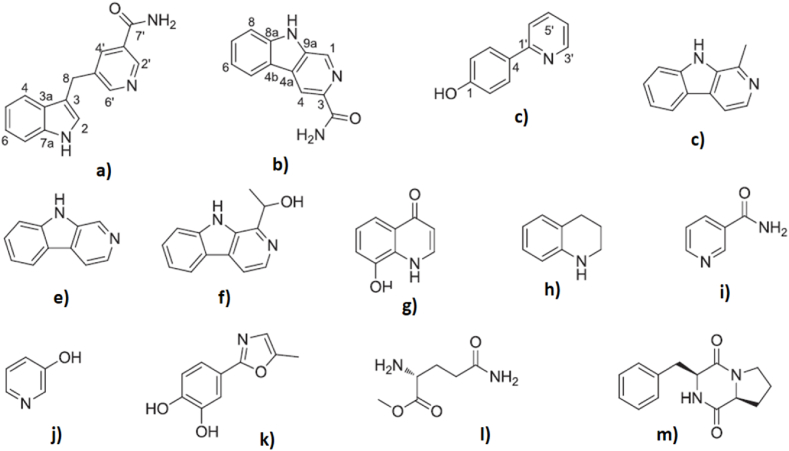
Structure of thirteen nitrogen containing, non-peptide substances extracted and isolated from the edible Chinese black ants (*Polyrhachis dives*). **a**) 5-(3-Indolylmethyl)-nicotinsaureamide; **b**) β-Carboline-3-carboxamide; **c**) 4-Pyridin-3-yl-phenol; **d**) harman; **e**) β-carboline; **f**) S-1-(1′-hydroxyethyl)-β-carboline; **g**) 8-hydroxy-4-quinolone; **h**) 1,2,3,4-tetrahydroquinoline; **i**) niacinamide; **j**) 3-hydroxypyridine; **k**) 2,5-disubstituted oxazole; **l**) glutamine methyl ester; and **m**) cyclo-(L-Pro-L-Phe). Source: [253].

The Chinese black ant (*Polyrhachis dives*) is an edible insect with kidney-detoxifying and antiinflammatory properties [253]. The ants contain several compounds essential for immunosuppressive, antinflammatory and renoprotective effects. Recently, several compounds were isolated from the species (Fig. 4).

**Fig. 5:**
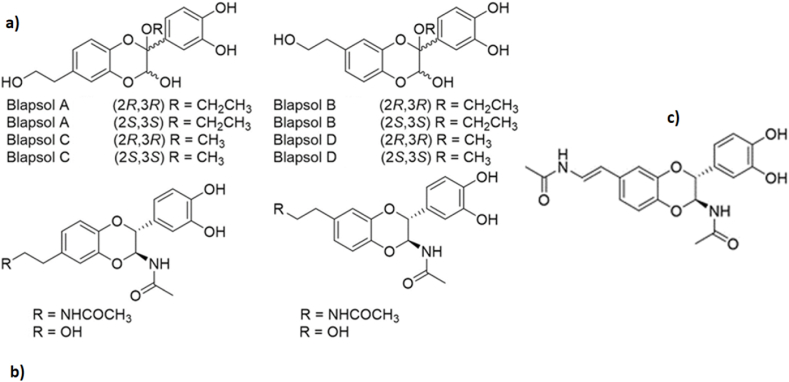
The blapsols **a**) and dopamine dimers (**b** and **c**) extracted from *Blaps japanensis.* Source [6].

The Chinese medicinal Insect *Blaps japanensis*, has been used to treat many diseases, including fever, cough, rheumatism, cancer, and inflammatory disorders. The species contains blapsols (Fig. 5a) and dopamine dimers (Fig. 5b and 5c). These chemical components have been evaluated for their effectiveness against cyclooxygenase (COX) enzymes COX-1 and COX-2. The enzymes catalyze the conversion of arachidonic acid to prostaglandins, which is useful in pain, fever, and inflammation [6,254,255].

The silkworm pupae are helpful to human health because of their high nutritional value and the many pharmacological effects they can have when consumed [73]. A vasorelaxant derived from the pupae of *Bombyx mori*, dimethyl adenosine, inhibits phosphodiesterase and stimulates nitric oxide production in endothelial cells, thus serving as a potential drug for treating vasculogenic impotence [22].

**Fig. 6:**
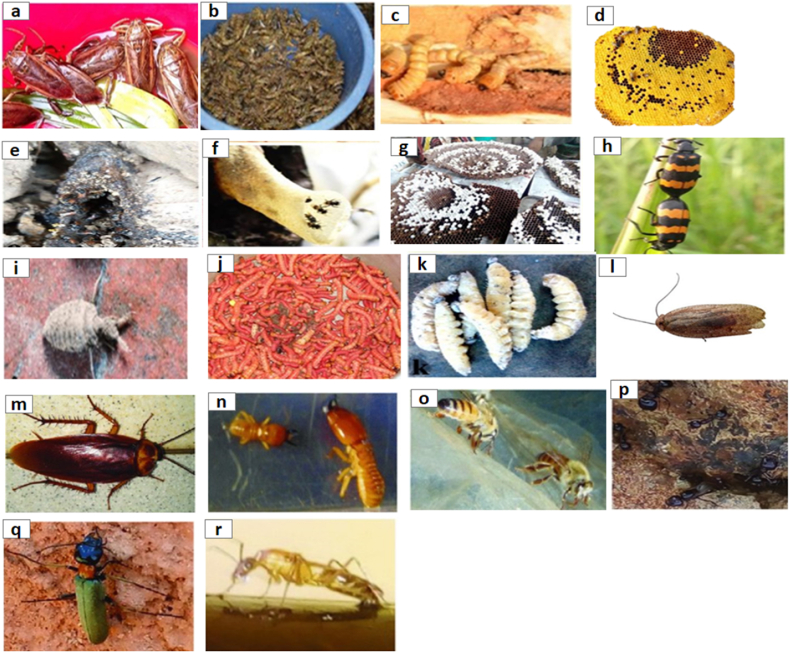
Certain medicinal insect and insect products of Nagaland (**a**–**i**) and Kadiogo and Houët provinces in Burkina Faso (**j-r**). **a)** Giant water bug *Lethocerus indicus*; **b)** dragonfly nymphs; **c:** large timber-boring larvae; **d)** freshly harvested *Apis florea* bee comb; **e-f)** nest entrances of stingless bees; **g)***Vespa mandarinia* comb sold at local market, Kohima district; **h)** blister beetle *Mylabris* sp.; **i)** larvae of antlion *Myrmeleon* sp.; **j)** larvae of *Cossus* sp.; **k)** larvae of banana skipper *Erionota torus*; **l)***Epilambra* sp. Cockroach, **m)***Periplaneta americana*; **n)***Macrotermes* sp.; **o)***Apis mellifera*; **p)***Pachycondyla* sp.; **q)***Lytta* sp.; **r)***Camponotus maculatus*. Source: [28,39].

Chinese, Korean, and Japanese acupuncture and traditional medicine practitioners have used bee venom (B·V.) to treat inflammatory illnesses by administering a sterile bee sting or injecting a prepared B.V. solution [256,257]. The use of insects and insect-derived products for disease treatment is substantially lower than typically claimed in Asia, Europe and Africa [258]. A study conducted in Kadiogo and Houet showed that about 19 insects belonging to 6 orders were important in treating about 78 different diseases and conditions, such as vomiting, headaches, deafness, pain and Inflammation [39]. Many insects are also used to treat different kinds of diseases in India [28]. The insects and insect-derived products used as medicine in India and Burkina Faso are illustrated in Fig. 6, in which the Giant water bug *Lethocerus indicus*, dragonfly nymphs, large timber-boring larvae, freshly harvested *Apis florea* bee comb, nest entrances of stingless bees, *Vespa mandarinia* comb, blister beetle *Mylabris* sp., larvae of antlion and *Myrmeleon* sp., are recorded from Nagaland (Fig. 6 a-i) and larvae of *Cossus* sp., larvae of banana skipper *Erionota torus*, *Epilambra* sp. Cockroach, *Periplaneta Americana*, *Macrotermes* sp., *Apis mellifera*, *Pachycondyla* sp., *Lytta* sp., and *Camponotus maculatus* are reported from Kadiogo and Houët provinces (Fig. 6 j-r) [28,39]. Although the Angami people of India employ the larvae of the banana skipper *Erionata torus* to treat dangerous animal bites, the Lotha people utilise them as an aphrodisiac. *Mylabris* sp. Is used to treat blisters and warts in India and are also included in the traditional Chinese medical pharmacopoeia and Korean medical pharmacopoeia. A study by Ouango et al. [39] showed that insects had been used in Burkina Faso to treat many diseases. The traditional healers in Burkina Faso utilise the cockroach *Periplaneta americana* (Fig. 6), to relieve ear pain. *Microtermi* spp. Is also used to treat diarrhea and fractures in Burkina Faso. Asthma, rheumatological pathologies, bladder lithiasis, burns, constipation, difficulty breathing, general fatigue, gynaecological problems, heart diseases, hip pain, insomnia, intestinal helminthiasis, and voice extinction are just some of the many conditions that can be helped by medicines derived from *Apis mellifera*. The bees are also used to reat female infertility and male impotence. The blister beetle *Lytta vesicatoria* is urinary track infection. Insects and insect-based substances have a long history of usage as food and feed in many parts of the world [259]. In many regions of the world, entomotherapy is used by various segments of society. In Northeast India, locals have identified twelve insect species as having medicinal value. These insects are being employed by the tribes and used to cure a wide range of illnesses in humans and domestic animals [260]. Coughs, fevers, nighttime emetic production, burns, and gastrointestinal illness were all treated with one of nine species found in Bangladesh [1,28].

### 4.1 Regulations of entomotherapy and entomophagy

Eating insects, or “entomophagy,” has evoked a wide range of feelings in people. Many psychological hurdles must be overcome before it can become mainstream because it is commonly held that neophobia and revulsion are the primary psychological factors that people use to reject entomophagy. With insects in medicine many illnesses have been treated with insects and insect extracts in folk medicine [261]. However, there are challenges associated with the consumption of insects and their therapeutic uses. Traditional medicine is still widely used in many parts of the world, including India, Korea, China, South America, and Africa. However, the practice of tradiational medicine has received little attention in Western culture and economically developed nations [6]. Zimmer [17] reported claimed that the Maya people have been utilizing maggots for therapeutic purposes for about 1000 years. These larvae consume decaying tissue which serves as a habitat for gangrene-causing bacteria that can cause health problems. In many African countries, there is limited access to modern medicine so traditional medicine, which frequently involves the use of insects, is nonetheless widely practiced in some parts of the continent [8,258].

### 4.2 Differences in entomotherapy between western countries and other regions in the world

Acceptance is still a barrier to the widespread use of insects as a medicinal resource for treating diseases and illnesses, especially in developed countries where most people view insects with distaste [262]. However, traditional medicine practices are widely accepted and documented in Chinese and Korean society, but less is known about similar traditions in Africa [263]. Traditional medicine, which sometimes includes insects, is nevertheless widely practised in parts of Africa where access to modern medicine is limited. This alternate medicine has largely received less attention since the advent of modern medicine, partly because of the baffling directions for various treatments [8,45]. As a result, entomotherapy, where insects are used to treat illness, is sometimes disregarded as superstition in many parts of the world. However, traditional medicine is still used in many parts of the world, such as India, Korea, China, South America, and Africa, despite being less popular in Western culture and economically developed nations [6,264]. It is believed that the general public in South America was more open to the concept of using insects as medicine because they were already using them as food in the past [8]. However, in Europe, therapeutic uses appear to have come before gastronomic ones [26,265]. Stick insects are used for treating calluses, warts and prickling spines in the Naga tribes, but in North Korea, they are considered to contain potent healing powers and are used to cleanse the body and remove stomach upset [266].

## 5 Production of the insects

### 5.1 Earlier principles of collecting the insects

A review of medical use of insects can at least date back to 2000 years ago when the book Sheng Nong's Herbal Classic described multiple medicinal applications of insects [267]. Till nowadays, only a few species are successfully mass produced [268]. More than 90% of the edible insects are collected from the field [[268], [269], [270]].

Insects for medicinal purposes differ from insects as food and feed for nutritional purposes in their exact medical effects requirements. Since the nutrients of insects vary even within species [271], principles of insect wide harvesting may include detail description of the stage, sex, time, location, and process to ensure their medical effects. For example, the *B. mori* adult used in medicine should be the newly emerged male with wings and legs removed [66]. Cicadidae periostracum should be collected in late summer and early autumn when cicadas newly emerged [66]. The best time to collect *Mylabris* sp. Beetles is the first month of autumn among the thorns of specific plants (e.g., *Sophora* sp.) [27]. *Rhynchophorus palmarum* (L.) (Coleoptera: Curculionidae) larvae collected from the native palm tree *Mauritia flexuosa* (L.f.) (Arecaceae: Arecaceae) is with the best healing properties [272].

Due to the uncertainty (e.g., quantity and quality across season and location) of wide harvesting, some insects have been domesticated for their commercial value. Insect domestication has at least 7000 years history [270], for example the honeybees and silkworms were domesticated during agricultural development [268]. Besides, crickets, mealworms, and the America cockroaches have been successively reared artificially. For example, a farm in Shandong, China, was reported to produce 20,000 kg of dry cockroaches annually [269]. However, not all the insects can be raised completely in artificial conditions. For example, locusts, wasps, and dragonflies are raised in a semi-domesticated way, which means part of the lifecycle is raised indoor or the nature habitat is manipulated to promote production [269]. Most of the rest of the medicinal insects are then still collected in the wide manually by local farmers.

Alternatively, if the effective ingredients have been identified, producing the specific ingredients rather than the insects would be a promising option. For example, the fermentation extract of mycelia from cultivated of *C. sinensis* is widely used in commercial sale with similar medical function, which has been an effective substitute of wide collection of infected ghost months, *Hepialus armoricanus* (Oberthür) (Lepidoptera: Hepialidae) [273].

### 5.2 Need for industrial production to produce large quantities of insect-based medicine

The need for insect-based medicine is increasing (Table 10). The emerged medical issues, for example, the increasing cases of cancer due to aging and antibiotic resistance problems leading to urgent requirements of novel drugs [274,275]. Therefore, medicinal insects, which can be potential sources for novel drug discovery, have gained increasing attention in the past decades due to their well-documented functions (e.g., anti-cancer and antimicrobe) [6]. Moreover, people are paying increasing attention to preventive medicine [276]. The willing of health and healing in daily life resulting in the debate of the concept of “food as medicine” [277]. To promote traditional medicine, World Health Organization (WHO) has established the global center for traditional medicine in India [278].

**Table 10:** Factors pull/push towards medicinal insect mass production.

The need for medicinal insect mass production	References
**Factors pull towards medicinal insect mass production**	
The need for novel drugs	[274,275]
The need for preventive medicines	[276]
The establishment of the WHO Global Center for Traditional Medicine	[278]
**Factors push towards medicinal insect mass production**	
Over-exploitation leads to species crisis and habitat destruction	[273]
Quantity variation	[279]
Quality variation	[76]
Safety concerns	[280]

The increased need for medicinal insects has led to overexploitation resulting in severe insect population crisis and habitat damage. For example, the *C. sinensis* infected ghost moth larva is an important anti-cancer Chinese medicine resource. However, the geographic distribution is confined, which is only available in soil of Qinghai-Tibet Plateau with 3500–5000 altitudes [273]. Overexploitation has pushed the local ghost moth larva facing extinction [273]. Another example is from the bamboo caterpillar, *Omphisa fuscidentalis* Hampson (Lepidoptera: Crambidae). The traditional harvesting activity usually cut down the entire bamboo clumps which is destructive [281].

Besides, the variation across season is a major challenge in commercialization of wild harvested insects. For example, in Republic of Congo, the migratory locust, *Locusta migratoria* (L.) (Orthoptera: Acrididae) is only available in November and December while the Termite is only available from November to next April [279]. Referring to the quality variation, modern research has determined many biotic and abiotic factors associated with quality variation. For example, the antimicrobe effects of honey have been determined to vary by species [282], geographical locations [76], types of flowers [225], and the age of honey [208].

More importantly, wild harvested insects have huge safety concerns. Heavy metal is a big concern nowadays due to civilization pollution. For example, the copper level of the wide harvested *Mylabris* sp. (Coleoptera: Meloidae), which used as an anti-cancer resource, was once determined reaching ∼45 mg/kg resulting in carcinogenic risk [283]. Pathogen contamination is another health risk. For example, pathogens (e.g., *Bacillus* sp. and *Staphylococcus* sp.) associated with foodborne disease have been determined in raw edible grasshoppers, *Ruspolia differens* (Serville) (Orthoptera: Terrigoniidae), in Uganda [284]. Besides, agricultural residues, for example veterinary drugs, antibiotics, and mycotoxins, found in wide harvesting insects become major biohazards in medicinal insect market [280].

### 5.3 What would mass production of insects look like?

Medicinal insects are only small parts of the beneficial use of insects. Besides bees and silkworms, the mass production of sterile screwworm, *Musca macellaria* Fabricius (Diptera: Calliphoridae) [285] on artificial diet for bio-control purpose is a milestone in insect mass production [270]. The edible insect has gained significantly increasing attention in the past decade as a nutrient pack for food and feed. Insects in general have higher feed conversion efficiency and lower environmental impacts compared to traditional livestock, which are believed to be one of the key solutions against food crisis [270]. Accordingly, the amount of investment, research, and company work on insect mass production increased significantly [268] after the Food and Agriculture Organization of the United Nations (FAO) recommended insects as food and feed in 2014 [286].

Though the nutrient and environmental requirements differ by species. There are following issues need to be considered before setting up an insect farm (Table 11). To set up a mass production farm is not as easy as a small-scale farm because the high density of insect and the subsequent issues related (e.g., metabolic heat and disease).

**Table 11:** Potential aspects to consider for medicinal insect mass production.

Aspects	Examples	References
Breeding	Genetic diversity, inbreeding depression, etc.	[280]
Feed source	Stable quantity and quality, cost efficiency, nutrient requirements, physical form, etc.	[287,288]
Facility	Location, logistic, mass and energy/heat balance, process-type, abiotic factors, remote sensing monitoring system, etc.	[[289], [290], [291]]
Processing	Harvesting, killing methods, decontamination, end product form, etc.	[292]
Packaging and storage	Lipid oxidation, re-moisturization, etc.	[293]
Insect disease	Virus, bacteria, fungus, mites, etc.	[294]
Hygiene and sanitation	IPIFF Guide on Good Hygiene Practices	[295]
Regulations	Novel food regulation	[296,297]

#### 5.3.1 Breeding

Now most of the edible insects are obtained by trading and a few are wide-collected and reproduced indoor, which the generic diversity is generally uncleared while the concept of insect breeding for food and feed is new [280]. Domestication is a gene selection process. Attention should be paid to insect industrialization, especially medicinal insects, to avoid inbreeding depression, effective ingredient reduction, and increasing vulnerability to pests and diseases [298]. For example, selection for silkworm cocoon weight trait after four generation resulted in poor survival rates [298].

#### 5.3.2 Feed source

The standard quality and continuous supply of feed is essential for insect mass rearing. Depending on the type of insect, the range of feed sources availability varies. For oligophagous like silkworms, the mass production of mulberries is required traditionally. In order to facilitate the sericulture, artificial diets for silkworms have been particularly developed [299]. Under the scope of edible insect production, omnivorous (e.g., crickets) insects are preferred and huge efforts have been put towards the organic waste stream exploitation and formulation [287,288] to meet the low-eco impact willing in insect farming. Besides feed source exploitation, the nutrient (species, stage, and age dependent) and physical form (mouthpart dependent) requirements [268] should be deeply studied to ensure a healthy and reproductive colony.

#### 5.3.3 Facility

The location, mass and energy/heat balances [289], modelling and simulation [290], logistic [291], and the process-type (e.g., batch and continuous systems) should be carefully considered ahead to ensure environmental control system (e.g., temperature, humidity, and ventilation) meet the insect requirements and the workflow is optimized. Life cycle assessment (LCA) [300] and hazard analysis and critical control points system (HACCP) [301] plus a remote sensing monitoring system would be helpful in dealing with such complex system and towards precision agriculture [302].

#### 5.3.4 Processing

Traditionally, most of the medicinal insects were sun dried and then boiled or fried before consumption [66]. Open and unhygienic drying conditions can cause microbes contamination [303]. Along with the development of edible insect industry, more processing methods were addressed, for example freeze drying, oven-frying, fluidized bed drying, microwave drying [292]. Further processing for protein, lipid, and chitin extraction can be achieved by pressing, ultrasound-assisted extraction, cold atmospheric pressure plasma, and dry fractionation [292]. As an alternative to drying, which is considered as an energy-consuming process, fermentation could be applied to raw insects [304]. While the above process would be enough for edible insects as food and feed, further processing (i.e., refining) may be required for medicinal insects processing to concentrate on the effective ingredients, for example the mass production of Xinmailong requires bioactive fraction extracted from *P. americana* [305].

#### 5.3.5 Packaging and storage

Lipid oxidation can generate toxic products, which are correlated with inflammatory diseases, cancer, atherosclerosis, and aging [306]. Oxidation is common during processing and storage especially for lipid-rich products like insects [293]. Antioxidants and vacuum-filling nitrogen packaging were determined to be a good method to avoid storage-phase oxidation [293]. Proper packaging and storage environment can also help in remaining low moisture content to suppress microbe growth [307].

#### 5.3.6 Insect disease

Mass production pushed insects to growth and develop at a high density, which provides optimal conditions for insect disease transmission [294]. For example, the *A. domesticus* densovirus (AdDNV) has caused mass mortality in cricket farms [308]. Hygiene is essential in insect farming to prevent disease transmission. However, once contaminated, shut down the production line and deeply clean the facility seems to be the only option in many cases [308]. Up to date, little is known about the insect pathogens, therefore a programmed called INSECT DOCTORS has been funded in Europe for insect disease specific research [309].

#### 5.3.7 Other

While the above aspects are more related to technical issues, other challenges (e.g., trained technicians, labors, and regulations) need to be overcome by the whole community (e.g., academia, industry, and the consumer society). For example, to avoid food-borne disease contamination, insect farms should follow some hygiene and sanitation protocol. A detail guide on hygiene practices of insect farming can be found on the website of international platform of insects for food and feed [295]. The guide covers legislative requirements from feed stream preparation to harvesting and processing. In European Union (EU) market, insects are viewed as novel food which must be approved following the Novel Food Regulation (EU) 2015/2283. Regulations on insects as food and feed has been reviewed by Ref. [297]. Regulations [296] though ensure consumers receive a good quality product; it usually takes a long time to come out. Therefore, to mass produce medicinal insects that are documented with specific effects, to synthesize the effect compounds, or to discover medicinal effects among approved edible insects, the discussion remains.

## 6 Future perspectives and conclusions

Insects have been widely used as medicinal resources in many parts of the world since ancient times. Insects can be used alone or combination with medicinal plants in the treatment of diseases [39]. The promotion and application of medicinal insects play a key role in all existing disease treatments. Though insects form part of the human diet in many countries and regions of the world, their use for medicinal purposes is often not promoted, and Western practice of entomotherapy seems dominant. A wide variety of insect species from different orders, such as Blattodea, Coleoptera, Hemiptera Hymenoptera, Lepidoptera, Odonata, and Orthoptera, contribute to the treatment of diseases in humans. Nevertheless, clinical trials assessing diseases' treatment through entomotherapy have received little attention than insects and insect-derived products utilized as food and feed. In the form of eggs, larvae/nymphs, adults and their derived products, insects provide alternative medicinal properties to modern medicine, though a few studies have attempted to address this issue in some countries. Even countries that use insects and insect-derived products have focused on a few geographical locations. As a result, global records on insect and insect-derived products for disease control are poorly documented worldwide, especially in Africa. More than 2100 insect species are eaten by humans in a wide variety of regions and countries, but little is known about the possibility of using these edible insects in the study and development of new medications and vaccines to battle disease. As a result, medicinal uses of insects, including treating diseases induced by pollution, microorganisms, allergens, and other higher animals, such as snakes and scorpions, could provide insight into the benefits of insects in treating emerging diseases and illnesses. Understating sustainable methods of rearing insects in medicine is critical for biodiversity conservation and prevention. However, insect farming is a concern regarding environmental issues and safety.

In effect, captive farming of edible insects can offer feed and food for animals and humans, respectively, and provide resources for the pharmaceutical industry to discover drugs for various health-related complications. Specifically, among others, future research should provide proper identification of these medicinal insects using molecular tools and conduct further investigation to verify and assess the viability of utilizing insects in the drug discovery process. Limited information exists on the reservations about entomotherapy as there are about entomophagy. Moreover, a clear understanding of the therapeutic use of insects and insect-derived products between Western countries and other regions worldwide requires further investigations. There is also a need to assess consumer opinions/consumer science on entomotherapy. Progress has been towards using insects for medicinal purposes. However, knowledge about their side effects is generally lacking. The relationship between disease treatment using insects and insect-derived products and allergenic effects in patients should be considered in future studies. While a clinical evaluation of medicinal plants’ efficacy and safety have been considered by several researchers, such information on medicinal uses insects is poorly documented. Future works should also investigate knowledge, perception, and willingness to apply and pay for entomotherapy. In addition, implications of using insects and insect-derived products on insect biodiversity conservation, the use of insects for the treatment of animal diseases and the contribution of insects to drug discovery may offer a new direction and solution to emerging diseases. Furthermore, among insects used for therapeutic purposes, some are pests responsible for diseases in plants and humans and others play a role in biological control as predators and pollinators of crops. In view for environment and biodiversity conservation, there is a need to select few samples of these insects in investigations necessary to ecological balance.

## Author contribution statement

**S.A.S.** – Conceptualization, Validation, Formal Analysis, Resources, Writing - Original Draft, Writing - Review and Editing, Visualization, Data Curation, Project administration, Supervision. **C.L.** – Writing - Original Draft. **O.F.A.** – Writing - Original Draft. **I.F.** – Conceptualization, Writing - Review and Editing. **M.A.H.** – Formal Analysis, Validation. **J.A.M.P.** - Review and Editing. **A.B.** - Data Curation. **A.G.** - Data Curation. **J.S.C.** - Review and Editing.

## Funding

This research was funded by FCT-Fundação para a Ciência e a Tecnologia through the CQM Base Fund - UIDB/00674/2020, and Programmatic Fund - UIDP/00674/2020, by Interreg MAC 2014–2020 Cooperacion territorial through AD4MAC project (MAC2/1.1 b/350), and by ARDITI-Agência Regional para o Desenvolvimento da Investigação Tecnologia e Inovação, through the project M1420-01-0145-FEDER-000005 - Centro de Química da Madeira - CQM+ (Madeira 14–20 Program) and the Post-Doctoral fellowship given to JAMP (Project M1420–09–5369-FSE-000001). The authors also acknowledge FCT and Madeira 14–2020 program to the Portuguese Mass Spectrometry Network (RNEM) through PROEQUIPRAM program, M14-20 M1420-01-0145-FEDER-000008).

## Conflict of interest

The authors declare no conflict of interest.

## Data availability statement

Not applicable.

## Declaration of competing interest

The authors declare that they have no known competing financial interests or personal relationships that could have appeared to influence the work reported in this paper.
